# Intraspecific microbiome dynamics across the life cycle of the milkweed bug Oncopeltus fasciatus

**DOI:** 10.1099/mgen.0.001583

**Published:** 2026-01-08

**Authors:** Will Larner, Nádia Thölke da Silva Grego, Kristen A. Panfilio

**Affiliations:** 1 School of Life Sciences, University of Warwick, Coventry, CV4 7AL, UK; 2 Department of Molecular Genetics, Institute of Biology, University of Hohenheim, Garbenstr. 30, 70599 Stuttgart, Germany

**Keywords:** Hemiptera, life history stages, microbiome, phytophagy, reproductive transmission, seed-feeding bugs

## Abstract

The microbiome is an important part of the complete nutritional and genomic profile of insects. The species-rich insect order Hemiptera (aphids, cicadas and true bugs) is highly diverse for mode of microbiome acquisition, with the conundrum that species in the seed-feeding subfamily Lygaeinae have lost obvious anatomy for housing bacteria, either in bacteriocytes or midgut crypts. Here, we characterize the microbiome of the milkweed bug *Oncopeltus fasciatus* as a tractable lygaeinid, using *16S rRNA* gene sequencing. We assess how bacterial taxa vary between the sexes and across life history stages in a controlled environment, focusing on maternal-to-embryo transmission and distinguishing egg-stage constituents that are superficial or internal (transovarially transmitted). Among a core microbiome of 28 genera, the egg stage shows the greatest diversity, with a particular expansion of the family *Comamonadaceae*. We also analyse inter-individual variability in nymphs and adults and validate structured, stage-specific detection of seed material. Comparative analysis identifies *Rhizobium* as a notable microbiome constituent in seed-feeding Hemiptera, which we had previously shown to lack nitrogen metabolism components in the genome. Overall, we provide a nuanced assessment of bacterial abundance dynamics between individuals and across the life cycle and discuss the implications for acquisition and potential relevance as nutritional endosymbionts. This will underpin comparative investigations in seed-feeding bugs and future work in *O. fasciatus* on tissue-specific and diet-specific microbiome profiles, including in natural populations.

**Impact Statement**

We provide a nuanced characterization of the microbiome in a model system insect for true bugs that are notable both for their diverse modes of microbiome acquisition and for their lack of evident microbiome-housing anatomy (the Lygaeinae). Given these ambiguities, our finding of the highest microbiome diversity at the egg stage, and that this is dynamic during embryogenesis with distinct eggshell surface and egg-internal constituents, highlights the value of vertical analysis across the life cycle. Equally, our post-embryonic sampling strategy reveals the extent of natural inter-individual variability in microbiome repertoires between the sexes as well as within a single nymphal instar. These findings are important for grounding future comparative studies across different populations and environmental niches. Lastly, in considering potential insect–bacteria nutritional endosymbioses, we identify nitrogen-fixing bacterial genera, some of which may be Hemiptera-specific, and link these to previous characterization of deficiencies in the host’s own genomic metabolic repertoire.

## Data Summary

The *16S* amplicon sequence raw data have been deposited in GenBank under the following accessions: BioProject PRJNA1213894, with 8 BioSamples (SAMN46351863-SAMN46351870, inclusive) and 48 SRA accessions (SRR32073024-SRR32073071, inclusive). SRA library names are identical to the sample identifiers throughout (e.g. Fig. 3C and the Data File S1). In detail: BioSamples: SAMN46351863 (EY), SAMN46351864 (EO), SAMN46351865 (EYW), SAMN46351866 (EOW), SAMN46351867 (Ni), SAMN46351868 (Np), SAMN46351869 (F), SAMN46351870 (M). And SRAs: SRR32073071 (EY1K), SRR32073070 (EY1R), SRR32073059 (EY3K), SRR32073048 (EY3R), SRR32073037 (EY4K), SRR32073028 (EY4R), SRR32073027 (EO5K), SRR32073026 (EO5R), SRR32073025 (EO6K), SRR32073024 (EO6R), SRR32073069 (EO7K), SRR32073068 (EO7R), SRR32073067 (EO8K), SRR32073066 (EO8R), SRR32073065 (EYW23K), SRR32073064 (EYW23R), SRR32073063 (EYW24K), SRR32073062 (EYW24R), SRR32073061 (EYW26K), SRR32073060 (EYW26R), SRR32073058 (EOW27K), SRR32073057 (EOW27R), SRR32073056 (EOW28K), SRR32073055 (EOW28R), SRR32073054 (EOW30K), SRR32073053 (EOW30R), SRR32073052 (N9K), SRR32073051 (N9R), SRR32073050 (N10K), SRR32073049 (N10R), SRR32073047 (N11K), SRR32073046 (N11R), SRR32073045 (N12K), SRR32073044 (N12R), SRR32073043 (N13K), SRR32073042 (N13R), SRR32073041 (F15K), SRR32073040 (F15R), SRR32073039 (F16K), SRR32073038 (F16R), SRR32073036 (F17K), SRR32073035 (F17R), SRR32073034 (M19K), SRR32073033 (M19R), SRR32073032 (M20K), SRR32073031 (M20R), SRR32073030 (M21K), SRR32073029 (M21R). All other data generated or analysed during this study are included in the manuscript and its supplementary information files, including nucleotide sequence data from Sanger sequencing in the Data File S1 tab ‘Sanger raw data’.

## Introduction

Insects are integral components of food webs and ecosystems across the globe [1,3]. With insect species’ abundances in flux as their geographical ranges change, in part linked to changes in local climate and human land use [4,5], understanding the dynamics of biodiversity has become a key challenge. One avenue to investigate this is to elucidate the genomic basis of insect feeding ecology, in terms of species’ capacities to exploit diverse or novel food sources or distinct ecological niches [6,9].

With >250 sequenced insect genomes to date [10], comparative genomics can reveal molecular evolutionary changes associated with ecology. This includes expansions, losses or rapid evolution in protein families with roles in environmental sensing (chemoreceptors and opsins), detoxification (antioxidants and antimicrobial peptides) and metabolism (digestive enzymes) [11,13]. For example, gene repertoire changes have been linked to dietary shifts between species [7] and between whole suborders of insects [14]. Mutations in specific metabolic enzymes underpin convergent adaptation by different species to the same host plant [e.g. 15,16].

Importantly, new genes acquired by lateral gene transfer from microbes [11,17, 18] or acquired directly by insects’ endosymbionts [19,20] are also associated with changes in insects’ food sources and geographic ranges. In contrast, the loss of gut microbiota correlates with impaired growth and fitness of the insect [21,22]. Thus, meaningful genomic profiling should encompass not only the insects’ own protein-coding repertoires, but also the extended genomic profile of the insect holobiont, including the microbiome [23,25].

 Integrated genomic profiling is particularly important for understanding the complex molecular basis of insect phytophagy, or plant feeding, strategies [14,19]. Phytophagy is widespread but also presents nutritional challenges. Food quality may be poor and lack essential vitamins or amino acids [20]. Plant tissues are protected by tough cell walls [17] and may contain toxic compounds such as cardenolides or other cardiac glycosides [15,16].

**Fig. 1.**
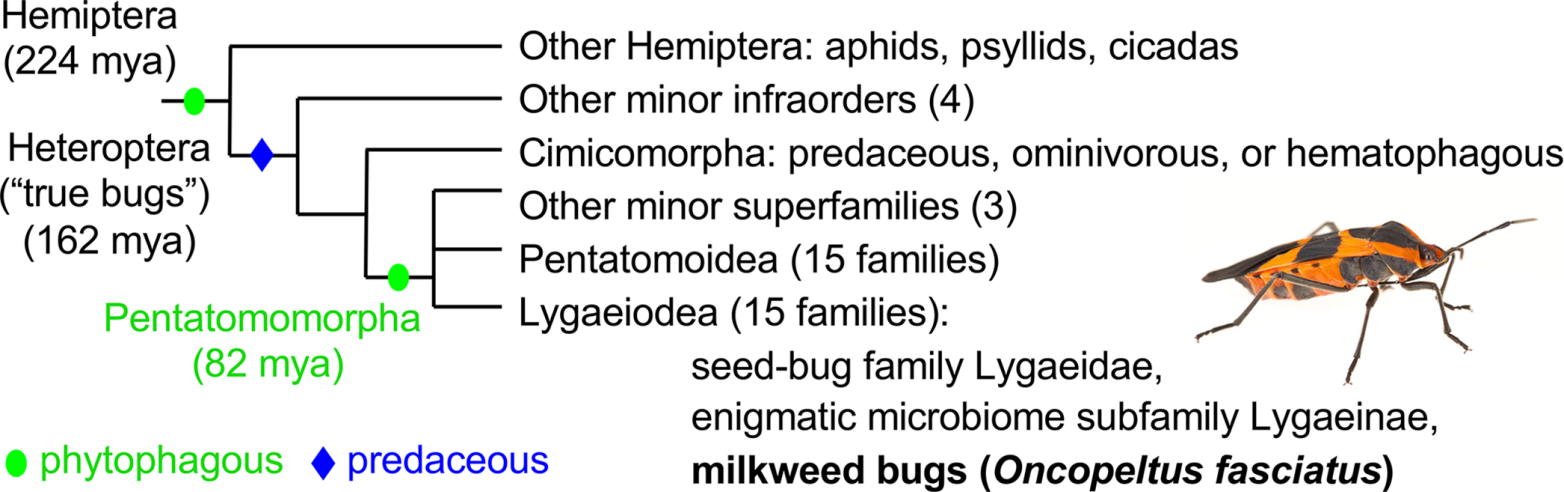
Feeding ecology, evolutionary history and phylogenetic context of *Oncopeltus fasciatus*. For the Lygaeinae, the designation ‘enigmatic microbiome’ refers to the absence of both midgut crypts and of bacteriocytes or bacteriomes as bacteria-housing structures that provide an anatomical framework for inferring microbiome constituents (i.e. extracellular or intracellular, respectively). Taxonomic relationships and feeding strategy changes: [9,26]. Milkweed bug image: Jena Johnson Photography, used with permission of the photographer, as in [11].

The Hemiptera is the most species-rich order of hemimetabolous insects, with members including aphids, psyllids and true bugs (Heteroptera) [26]. Secondary reacquisition of phytophagy in the true bug infraorder Pentatomomorpha (Fig. 1) [26] has led to radiations of species that are invasive and polyphagous [18,27], preferentially specialist [28] or strictly monophagous [29] for plant species and tissue. How do feeding ecology types compare for the metabolic repertoires of the insect itself or its microbial constituents?

Nutritional endosymbionts have been characterized in many Hemiptera [reviewed in 30,31], including prominent examples such as aphids, with a nutrient-poor, fluid-feeding lifestyle [20,32]. Within the true bug infraorder Pentatomomorpha, there is extensive diversity for endosymbiont transmission type, dedicated host anatomy and microbe taxonomy [29,33]. Briefly, bacterial acquisition is often environmental, through nymphal feeding at each generation [34], primarily via ingestion of faeces (coprophagy) from conspecific individuals [35]. Another predominant route of symbiont transmission is via hatchling ingestion of bacteria vertically provided by the mother, such as through egg smearing or capsule or jelly secretions external to the eggs [36,37]. Vertical transmission can also be transovarial, with bacteria deposited by the female directly into the egg [38]. Within the insect’s abdomen, bacteria may be harboured in dedicated midgut crypts, which is typical for extracellular *Gammaproteobacteria* in Pentatomoidea [39]. Alternatively, intracellular *Burkholderia* symbionts in Lygaeoidea are housed in bacteriocytes that are often distinctively pigmented and located near the gonads [40].

The seed-feeding subfamily Lygaeinae is a notable exception, with specialist and monophagous feeders with an enigmatic, understudied microbiome (Fig. 1). This subfamily lost midgut crypts, but reports differ for bacteriocyte presence, even within a genus [29,33]. Older sequencing analyses suggest these insects have a diverse microbiome [29,33, 39], and this awaits full characterization. The milkweed bug *Oncopeltus fasciatus* is a specialist feeder and research model for physiology, development and evolutionary ecology since the mid-twentieth century [reviewed in 11, 28, 39]. Adult anatomy and embryology are well described, corroborating the absence of obvious microbiome-housing tissues [41,44]. Although there has been long-standing interest in maternal nutritional provisioning of eggs [42,45], extensive embryological work – including direct observation of oviposition and handling of eggs – has found no evidence of substances deliberately deposited on the eggshell in this species [43,46, 47, cf., 48]. Furthermore, genome analysis identified an extensive metabolic enzyme repertoire, but with some notable omissions, such as in nitrogen metabolism and amino acid synthesis [11].

What is the composition of the *O. fasciatus* microbiome, and what proportions of the full complement derive from distinct acquisition strategies at different life history stages? Here, we characterize the microbiome profile across the life cycle, using amplicon sequencing of the *16S rRNA* gene. Our sampling focused particularly on maternal and embryonic stages, and we distinguished microbial constituents within the egg or on the eggshell surface. Furthermore, to control for potentially distinct ecological microniches between embryonic, nymphal and adult stages [28], and for precise assessment of inter-individual variability [49], we assayed bugs from a uniform laboratory colony environment. We hypothesized that the *O. fasciatus* microbiome is primarily acquired via transovarial transmission, as in other Lygaeoidea [29,33], slightly augmented through post-embryonic environmental acquisition [30,50]. In fact, we find high inter-individual nymphal variation and impoverished but sex-specific adult profiles, contrasting with diverse egg microbiomes with both transovarial and external taxa.

## Methods

### Milkweed bug culture and life history samples

A laboratory colony of *O. fasciatus*, cultured continuously since June 2014, was used (Carolina Biological Supply strain, Burlington, NC, USA; as used in, e.g. [8,11, 51]). Generational cohorts of bugs were maintained in plastic cages at 25 °C, with a 12:12 h light/dark cycle, and provisioned with unchilled filtered drinking water, peeled organic sunflower seeds (Holland and Barrett, Nuneaton, UK) and loose cotton wool for oviposition (Robinson Healthcare, Worksop, UK).

**Fig. 2.**
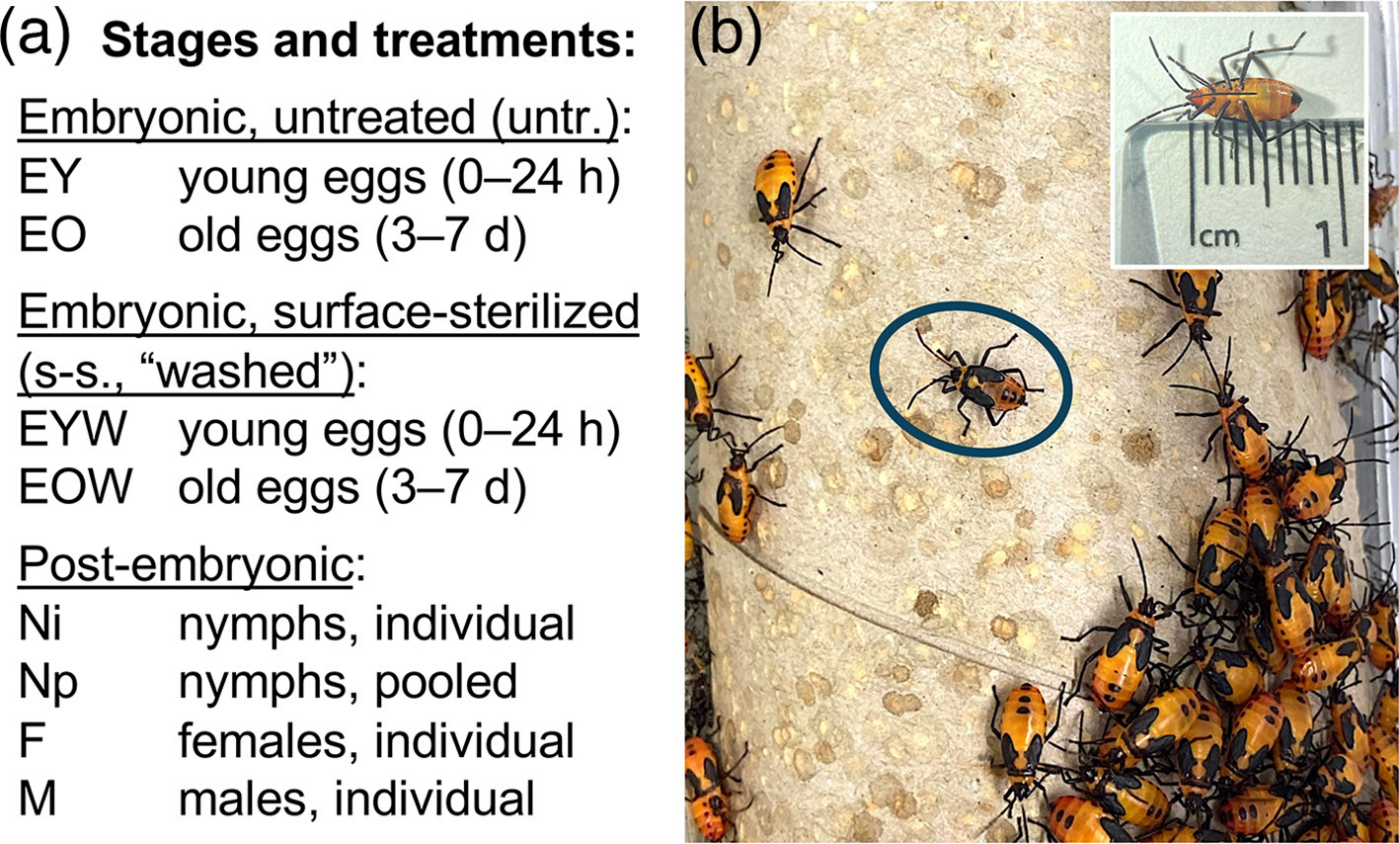
Life history stages and treatment conditions for microbiome profiling. (**a**) All treatment conditions are listed, including the ages of the embryonic samples and which post-embryonic samples were based on single individuals or pooled samples (three bugs were used in the pooled nymph samples). Analysed nymphs were from early in the fifth (final) juvenile instar. (**b**) Images of fifth-instar nymphs. Nymphs used in this analysis were <8 mm in length (inset) and thinner than late-instar nymphs close to the adult moult (compare the circled early-instar nymph with others in the cohort). The bugs congregate on a cardboard tube provided for spatial structure within the cage. The light and dark brown marks on the cardboard are from the bugs’ faeces, a standard feature of the colony environment.

 Seven life history stages and egg treatments were analysed (Fig. 2a). Embryonic samples were collected within the first day (young eggs, EY, 0–24 h) or the second half of embryogenesis (old eggs, EO, 3–7 days; precisely 70–165 h) and assayed as untreated eggs that retained external bacterial constituents (EY, EO) or after surface sterilization (washed eggs: EYW and EOW) to retain only egg-internal constituents. Nymphs (N) were assayed at the final (fifth) juvenile instar [52], as in previous true bug work [39]. Within this cohort (23–29 days), younger nymphs were selected by size for consistency (<8 mm body length, Fig. 2b). Reproductively active adults (2–4 weeks after the final moult) were sexed by abdominal morphology and pigmentation [41,53] for the male (M) and female (F) samples.

 Material for all biological replicates of a given sample type was collected on the same day from the same cohort; material for the different sample types was taken from different cages and generational cohorts, with independent DNA extraction for all seven sample types.

### Tissue preparation and DNA extraction

For embryonic stages, to avoid contact with the surface of the eggs, gloves were worn while extracting eggs from the cotton using a fine paintbrush and lightweight forceps. Eggs for the different biological replicates were evenly distributed among sterile 1.5-ml tubes, and dry weight was determined. Surface sterilization followed a modified protocol (after [39,40, 54, 55]): eggs were washed in ethanol (for >1 min each: once in 100% ethanol and twice in 70% ethanol/ddH_2_O) followed by three 2 min rinses in dH_2_O.

Nymphs and adults were anesthetized using CO_2_ and dissected under tap water in a sterile glass dish using sharp, ethanol-sterilized forceps. For nymphs, the legs were removed, and tissue was extracted by squeezing the posterior abdomen. For adults, the legs, wings and abdominal exoskeleton were removed, ensuring intact gut material was collected. Using the forceps, tissue pieces were lifted out of the dissecting dish, and excess liquid was shaken off before transferring material to a sterile 1.5-ml tube. Total tissue weight was thus determined in minimal residual liquid (range: 13.5–33.2 mg per insect; see also Data File S1, tab ‘Biomass and yields’, available in the online Supplementary Material). Adult samples were processed with one individual per biological replicate. For the smaller nymphs, replicates of either individual nymphs (Ni) or pooled samples of three nymphs (Np) were processed.

Additionally, environmental sampling was performed on laboratory food stocks. The sunflower seeds, crushed in a mortar and pestle, and milled wheat flour (Wright’s Bounty Premium Bakers White Bread Flour, Wright and Sons, Harlow, UK) were weighed to an input of 25 mg.

All samples were processed using the Quick-DNA/RNA Miniprep Kit (Zymo Research, Orange, CA, USA), with elution in the provided sterile water. DNA quality and quantity were confirmed by spectrophotometry, including multiple water samples as negative controls (Implen NanoPhotometer N60/N50, Munich, Germany). At subsequent steps, sample quality and concentration were determined with a TapeStation (Agilent, Santa Clara, CA, USA) (after sequencing library PCR synthesis) and with the AccuBlue High Sensitivity dsDNA Quantitation Kit (Biotium, Fremont, CA, USA) (final library concentration).

### *16S rRNA* gene analyses: amplicon sequencing and taxonomic classification

As in prior insect microbiome characterization [55], an established Illumina sequencing pipeline was followed (‘16S Metagenomic Sequencing Library Preparation’, version 15044223 Rev. B). Briefly, a ~425 bp portion of the *16S rRNA* gene was amplified with slightly degenerate primers targeting the V3 and V4 hypervariable regions (forward primer: 5′-tcgtcggcagcgtcagatgtgtataagagacagCCTACGGGNGGCWGCAG-3′, reverse primer: 5′-gtctcgtgggctcggagatgtgtataagagacagGACTACHVGGGTATCTAATCC-3′; adapter sequence in lowercase, *16S*-specific sequence in uppercase). PCR was performed according to the protocol, modified to include technical replicates with REDTaq Ready Mix (‘R’, Sigma/Merck, Darmstadt, Germany) or KAPA Taq EXtra HotStart ReadyMix PCR Kit (‘K’, Kapa Biosystems, Wilmington, MA, USA). Amplification for the ‘R’ replicate was performed in independent PCR experiments for all seven sample types, preventing cross-contamination. All ‘K’ replicates were performed in an independent PCR experiment from the corresponding ‘R’ replicate, except for surface-sterilized egg samples (EYW and EOW) where both technical replicates were amplified in parallel. Agencourt AMPure XP PCR purification with magnetic beads (Beckman Coulter, Brea, CA, USA) was used.

For three to five biological replicates per sample (two technical replicates each), subsequent library preparation and sequencing were conducted at the Cologne Center for Genomics. Libraries were prepared with the NEBNext High-Fidelity 2 × PCR Master Mix (New England Biolabs, Ipswich, MA, USA) and the Nextera XT Index Kit (Illumina, San Diego, CA, USA). Library preparation included a no-template (water) PCR negative control, with a negligible resulting DNA concentration and no visible amplicon band (TapeStation analysis), whereas all 48 samples were successfully synthesized (Data File S1, tab ‘Biomass and yields’). Sequencing was conducted with an Illumina MiSeq machine, using the v.3 kit with 2×300 bp reads, and recovered 14.6% PhiX-aligned reads, consistent with input levels of 10–15% for this spike-in sequence diversity control (PhiX Control v.3 phage genome library).

Reads were taxonomically classified with Illumina’s 16S Metagenomics app v.1.1.0, through the BaseSpace implementation of the Ribosomal Database Project TRDPU Classifier ([56]; version as of 7 October 2022). Raw reads were demultiplexed, and FASTQ files were generated to assess read quality. For classification, reads were identified through cumulative subsequence nucleotide similarity mapping to the RefSeq RDP *16S* v.3 reference taxonomy database, incorporating reference sequence data from silva version 132 [57], available from [58]. This includes distinct reference sequences for >50 phyla, >2,000 genera and >6,000 species (see Data File S1, tabs ‘Phylum (%, counts)’, ‘Genus (%, counts)’ and ‘Species (counts)’ for full details).

As an empirical, independent sequencing and classification approach, a selected dataset was Sanger-sequenced (as in [59]). For each biological sample type analysed in the main Illumina dataset, three to six clones were sequenced from each of the two technical replicates for the selected sample. Also, environmental sampling from insect food sources maintained in the laboratory (sunflower seeds and milled flour) was analysed, with new PCR amplification and additional sequencing of EO samples as positive controls. Amplicons were cloned into the pGEM-T Easy vector in JM109 competent cells (Promega, Southampton, UK), and M13 vector primers were used for sequencing (M13 forward: 5′-TGTAAAACGACGGCCAGT-3′, M13 reverse: 5′-GGAAACAGCTATGACCATGA-3′). After trimming the *16S* primers, clones were classified to the genus level through a consensus approach (as in [59,60]), based on outputs from silva [57] (sina Aligner v1.2.1 [61], to five silva-hosted reference databases) and blastn to three GenBank databases (both sites last accessed 28 July 2025). We found genus-level classification to be robust and commensurate with the level of nucleotide discrimination possible within the V3–V4 region of the *16S rRNA* gene [62] (see Note 1 of Table S1 for a detailed account and appraisal of classification rigour and caution).

### Statistical data handling and phylogenetic analysis

Total genus-level classified read data were used as inputs for unsupervised hierarchical clustering analyses (Partek Flow software, version 10, release 10.0.22.1003, Illumina). Briefly, per-sample data were represented by a normalized classification vector, which was then used to calculate a Pearson correlation distance matrix. Dendrogram visualization was then generated with the unweighted pair-group method with arithmetic mean (UPGMA) hierarchical clustering.

For the genus diversity profiles and for the Shannon Species Diversity Index, all bacterial taxa were considered. A threshold of 1% abundance was used for genus presence/absence, taken as the mean of the biological replicates, from the median of the two technical replicates. The threshold for species presence was ≥10 reads in both technical replicates for ≥1 biological sample.

Graphical and statistical analyses were conducted in GraphPad Prism (v.10.2.3–10.3.1), including tests for normality and determining the applicability of Welch’s correction for parametric pairwise comparisons. Maximum likelihood phylogenetic trees were generated with default settings of the phylogeny.fr analysis pipeline [63].

As sample handling can introduce bacterial contamination, such as from DNA extraction kits [64,67], we assessed data quality with the *decontam* statistical classification procedure [68] (R package version 1.28.0), using the frequency-based method. Of all 2,012 classified genera, only 4 were flagged as potential contaminants (0.20%, comprising 0.20% of all genus-level classified reads), and these only occurred at low abundance (≤0.13% each, mean of all samples). Similarly, at the species level, these potential contaminants represent a very minor fraction (0.11% reads and 1.31% species). The 34 genera that we discuss in detail in the ‘Results’ and ‘Discussion’ sections all substantially exceed the cutoff threshold for comprising genuine microbiome constituents (see Data File S1, tabs ‘Biomass and yields’ and ‘Contaminant assessment’, and Fig. S1 for further details on contaminant-calling parameters and assessments). As we have strong clustering of technical replicates (see first the ‘Results’ subsection), indicative of low-noise data [68], with only very few, low-abundance potential contaminants, we report our taxonomically classified read data in full, in line with recommendations for dataset transparency [67], with the cautionary note that the genera *Corynebacterium*, *Dermacoccus*, *Enhydrobacter* and *Rothia* may be contaminants not specific to the *O. fasciatus* microbiome.

## Results

### Dataset quality and a general trend of declining microbial diversity across the life cycle

**Fig. 3.**
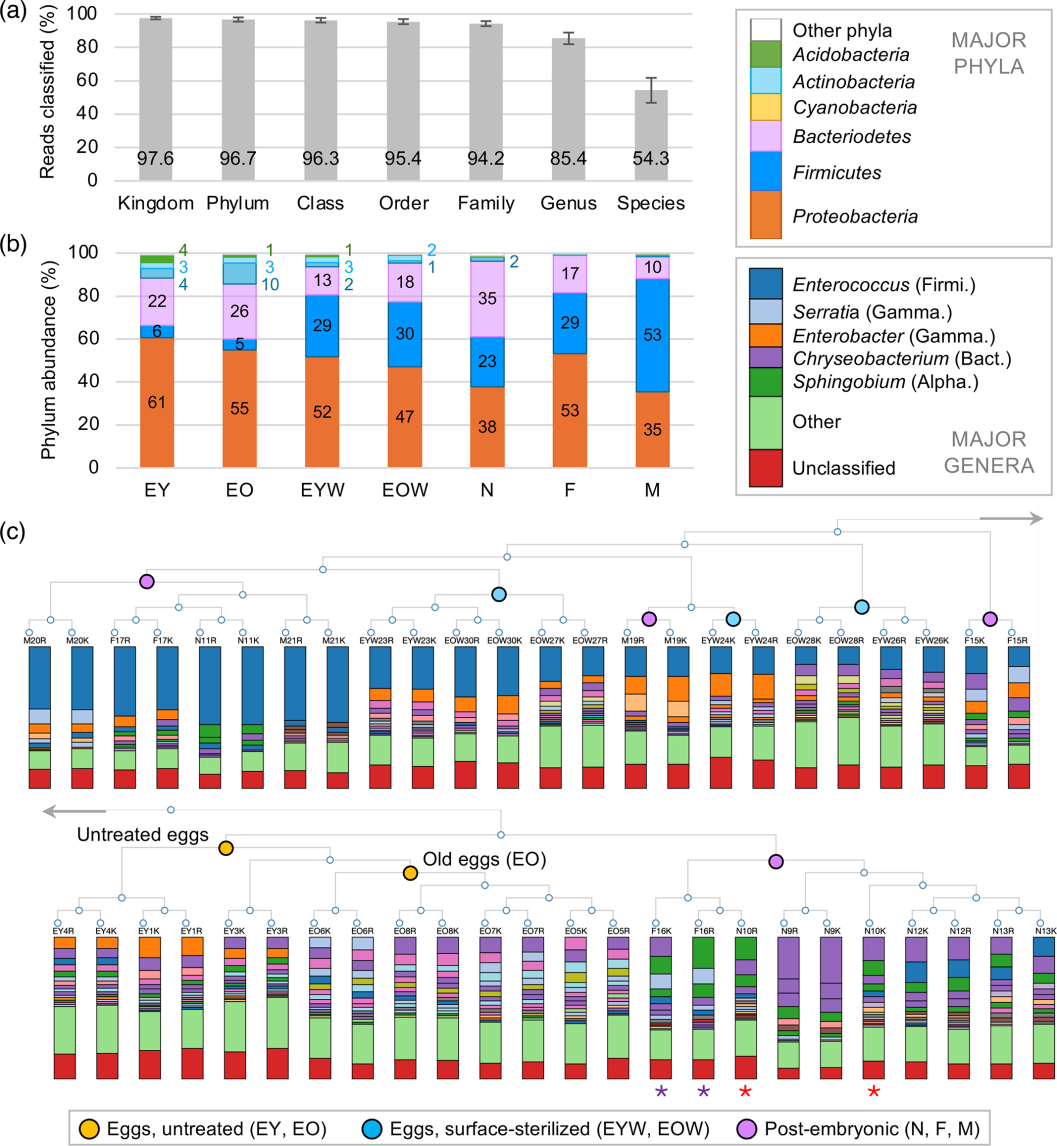
Overview of *16S rRNA* gene taxonomic classifications. (**a**) Proportion of reads classified per taxonomic level, shown as mean±sd from all biological and technical replicates (*n*=48). The vast majority of reads could be classified, albeit with a noticeable drop-off in classification efficiency at the species level. (**b**) Relative abundance (% reads) of the six most prevalent phyla (upper colour legend). (**c**) Genus-level hierarchical clustering dendrogram of all samples (see the ‘Methods’ section), with abundant genera from the top three phyla indicated (lower colour legend: parenthetical phylum and class affiliations are abbreviated as in Table 1). To accommodate all 48 samples, the dendrogram is split into the 2 major clusters, which join as indicated by the grey arrows. Coloured nodes indicate major clusters for untreated eggs (light orange: with a sub-cluster for old eggs), surface-sterilized eggs (blue: three clusters) and post-embryonic samples (purple: four clusters). Asterisks flag the sole instances where technical replicates do not strictly cluster (red, nymphal sample; purple, female sample). Life history treatment abbreviations are as in Fig. 2.

To characterize the milkweed bug microbiome across life history stages (Fig. 2), we first assessed data quality with respect to taxonomic level (Fig. 3a–c). We generated 13.35 Gbp of raw data for 61.1 M reads, with >110,000 classified reads per sample (*n*=48). On average, >94% of reads were classified to the family or higher level, declining at the genus and species levels (Fig. 3a). Nonetheless, we observed no correlation between read depth and species diversity (linear trendline fit *R*^2^ <0.29 in all cases: Data File S1, tab ‘Read depth statistics’), suggesting sufficient sequencing depth was obtained. Further, the technical replicates were congruent, with only two minor exceptions in genus-based clustering (Fig. 3c, asterisks). Thus, we integrated multiple taxonomic levels to strike a balance between robust read classification and taxonomic resolution.

**Table 1.** The major prokaryotic genera detected across the life cycle in *O. fasciatus* Twenty-eight genera were detected above the presence threshold of >1% abundance in at least one of the four major life history samples, and taxa are categorized by the number of life history samples in which they are present. For samples with <1% abundance, the exact value is specified, but these values are shaded. Phylum abbreviations: *Alpha*/*Beta*/*Gamma*, *Proteobacteria* of the indicated class; *Bact*, *Bacteroidetes*; *Cyano*, *Cyanobacteria*; *Firmi*, *Firmicutes.*

Taxonomy		Egg, untr.		Egg, s-s.		Nymph	Adult		Distribution
Phylum	Genus	EY	EO	EYW	EOW	N	F	M	
*Bact*	*Chryseobacterium*	16.61%	18.19%	8.97%	9.64%	28.70%	15.02%	3.16%	Present in all
*Alpha*	*Sphingobium*	7.13%	2.42%	1.23%	2.26%	15.18%	14.61%	1.18%	
*Firmi*	*Enterococcus*	3.38%	3.40%	28.08%	29.62%	20.15%	28.56%	49.47%	
*Beta*	*Delftia*	2.93%	3.50%	4.11%	2.34%	2.28%	2.23%	0.84%	
*Bact*	*Sphingobacterium*	1.90%	2.90%	3.33%	5.91%	1.96%	1.33%	0.97%	
*Gamma*	*Acinetobacter*	9.83%	16.38%	6.91%	7.95%	0.69%	0.18%	2.69%	Present in three (eggs and one post-embryonic stage)
*Gamma*	*Enterobacter*	9.61%	0.69%	13.29%	8.48%	0.03%	6.88%	10.45%	
*Cyano*	*Streptophyta*	4.99%	10.51%	2.23%	1.10%	1.95%	0.07%	0.46%	
*Gamma*	*Kosakonia*	3.66%	0.25%	1.76%	2.45%	0.01%	2.92%	0.03%	
*Bact*	*Flavobacterium*	1.99%	2.59%	1.33%	1.59%	2.91%	0.44%	0.18%	
*Bact*	*Dyadobacter*	1.84%	1.02%	0.36%	1.11%	0.12%	1.13%	0.52%	
*Gamma*	*Stenotrophomonas*	1.75%	0.63%	1.46%	0.87%	3.18%	0.18%	0.73%	
*Gamma*	*Klebsiella*	1.42%	0.10%	0.85%	1.13%	0.00%	1.17%	0.19%	
*Other*	*Acidobacteria (Gp15*)	4.04%	1.29%	1.29%	0.60%	0.25%	0.06%	0.19%	Present in two
*Gamma*	*Pseudomonas*	1.60%	5.46%	2.18%	3.44%	0.24%	0.13%	0.33%	
*Firmi*	*Staphylococcus*	0.93%	0.51%	2.09%	0.95%	0.81%	0.03%	3.27%	
*Alpha*	*Sphingomonas*	0.56%	0.28%	0.14%	0.29%	1.20%	1.06%	0.12%	
*Alpha*	*Brevundimonas*	0.41%	2.42%	1.28%	0.77%	0.83%	0.16%	0.07%	
*Alpha*	*Rhizobium*	0.13%	0.23%	2.44%	0.81%	1.11%	0.35%	0.35%	
*Gamma*	*Serratia*	0.12%	0.50%	2.78%	1.94%	0.00%	11.50%	5.76%	
*Beta*	*Xenophilus*	4.57%	9.73%	0.05%	0.73%	0.27%	0.30%	0.47%	Present in one
*Beta*	*Comamonas*	1.37%	0.89%	0.43%	0.47%	0.19%	0.21%	0.32%	
*Bact*	*Epilithonimonas*	1.05%	1.03%	0.10%	0.14%	0.11%	0.03%	0.01%	
*Bact*	*Nubsella*	0.90%	0.82%	0.41%	0.59%	1.62%	0.49%	0.06%	
*Beta*	*Pseudorhodoferax*	0.79%	1.32%	0.01%	0.09%	0.04%	0.04%	0.18%	
*Alpha*	*Shinella*	0.16%	0.54%	0.93%	0.21%	0.25%	0.10%	1.57%	
*Bact*	*Sediminibacterium*	0.12%	0.00%	0.00%	0.00%	0.08%	0.01%	6.19%	
Other	*Peredibacter*	0.05%	0.45%	0.04%	0.30%	2.12%	0.09%	0.01%	

 Overall, six bacterial phyla account for >98.7% of the *O. fasciatus* microbiome (Fig. 3b). The *Proteobacteria*, *Firmicutes* and *Bacteroidetes* are the most abundant (86–99%) and present in all life history samples. These phyla are predominantly represented by just a few major genera (Fig. 3c). Additionally, embryonic – and to a lesser degree nymphal – samples harboured >1% each of the phyla *Cyanobacteria*, *Actinobacteria* and *Acidobacteria*.

**Fig. 4.**
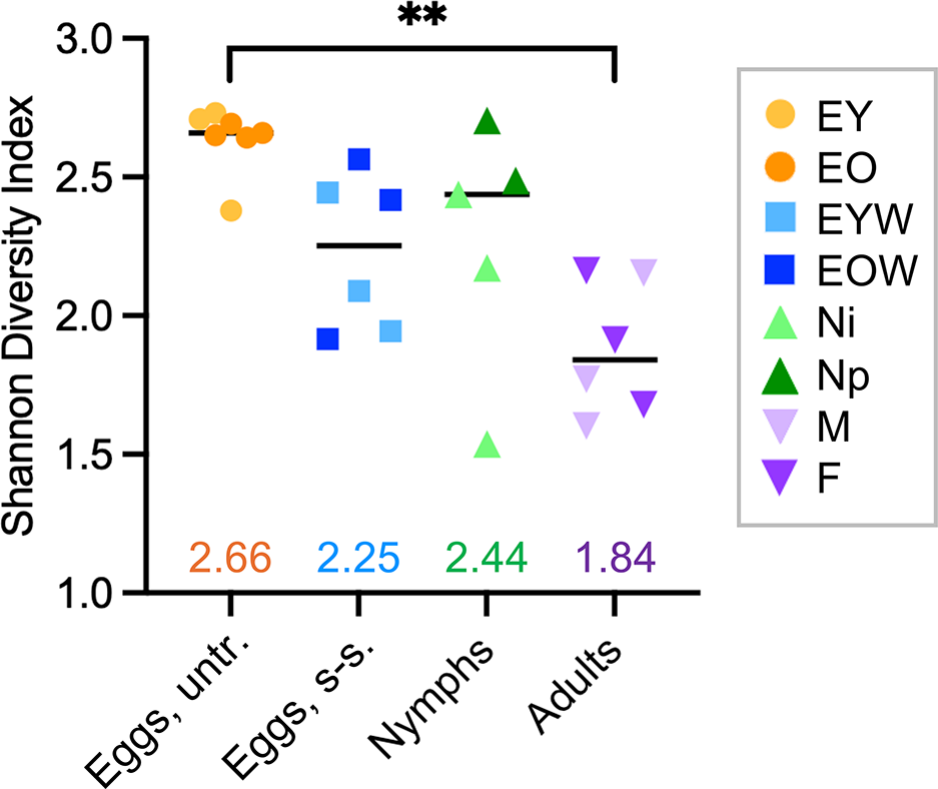
Diversity of the microbial community is greatest in untreated eggs. Shannon Diversity Index of bacterial species composition (accounting for species number and relative abundance) plotted for each biological replicate of the four major life history treatments analysed. Median values are indicated by the horizontal black bars and specified in coloured text. Light and dark plot points distinguish subcategories, as indicated in the legend (abbreviations as in Fig. 2). Significance was determined by Kruskal–Wallis and Dunn’s test, with the sole significant difference between untreated eggs and adults (alpha = 0.05, *p*_adj_ = 0.0021).

 These phylum-level trends are borne out at the genus and species levels for patterns of bacterial diversity and within-sample variation. Untreated eggs that retained surface bacterial constituents, particularly at the older embryonic stage, were the most similar across biological replicates (Fig. 3c, single node in the dendrogram for all untreated egg samples) and the most microbially diverse (Fig. 4, highest median value). Adult samples had the lowest median diversity of all sample types, with significantly lower microbial diversity than untreated eggs (Fig. 4). For the genus profile, most nymphal samples (four of five biological replicates) clustered with the untreated egg samples (Fig. 3c, lower half of dendrogram). In contrast, all surface-sterilized egg samples (six of six biological replicates) were more similar to most adult samples (five of six biological replicates), but with no clustering by life history stage (Fig. 3c, upper half of dendrogram). For life history samples derived from a single individual (*n*=3 per stage), nymphs exhibited greater variability in microbial diversity than either adult males or females (Fig. 4). Overall, this suggests that the egg (surface) is a uniquely diverse microniche, but what is the core microbiome profile, and which bacterial taxa account for major life history stage differences?

### The core milkweed bug microbiome is phylogenetically diverse

**Fig. 5.**
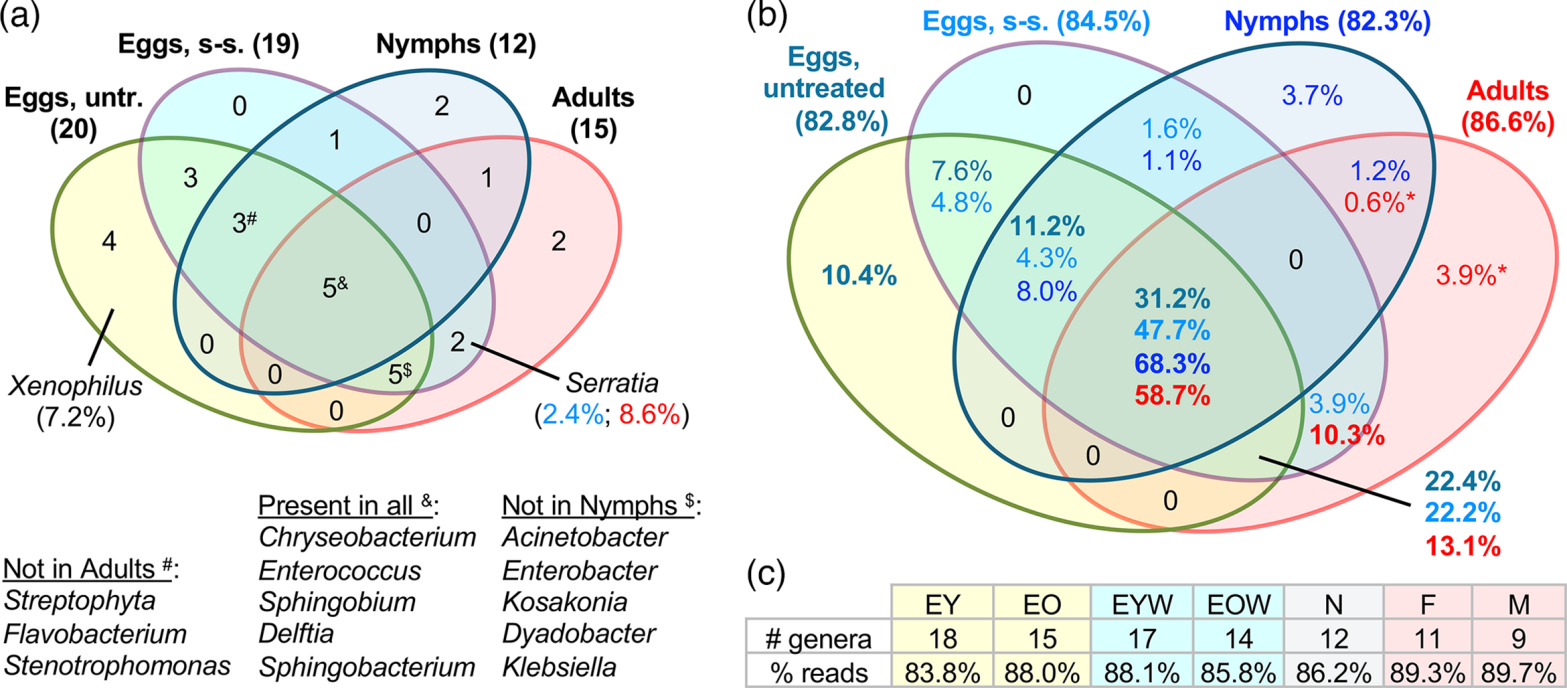
Distribution across life history samples of the 28 major genera of the *O. fasciatus* microbiome. Genera with >1% reads in at least one of the four major life history treatments are included. Venn diagrams are for (a) the number of genera and (b) the proportion of reads classified at the genus level. For diagram sectors with >10% reads, the genera are named, with selected abundances (% reads) specified parenthetically in (a). In (b), asterisks for low abundance in adult sectors reflect the result of averaging disparate abundance values between males and females (see also Fig. 7e). (c) For clarity, the genus counts and % reads are given for all seven individual stages and treatments. Note that unclassified reads, as well as genera with <1% abundance, account for ~15% of reads not documented here (cf., Fig. 3a, c).

**Fig. 6.**
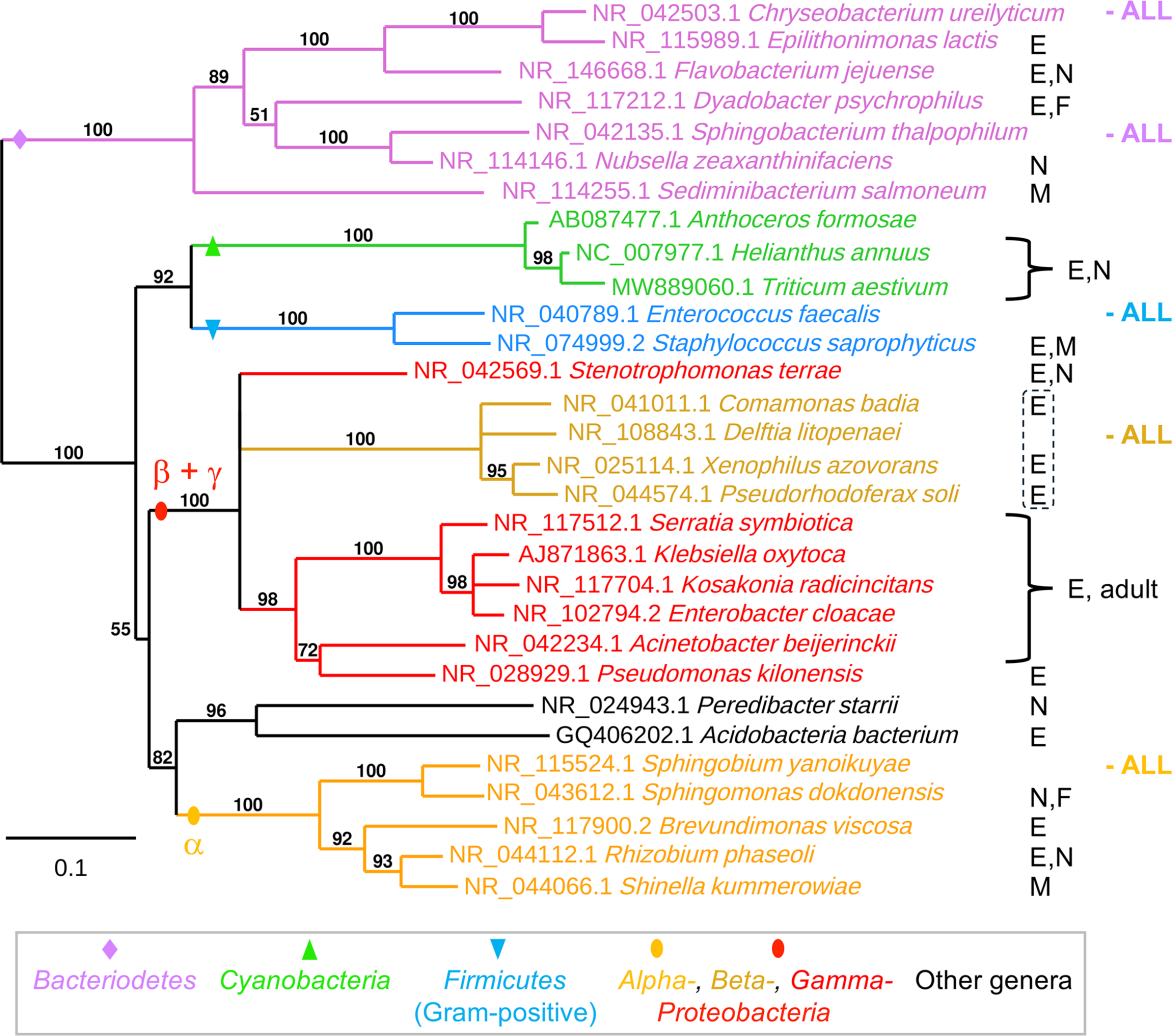
Phylogenetic distribution of the main microbiome genera. Maximum likelihood phylogeny for a >1,350-bp region of the *16S rRNA* gene (species names and GenBank accessions as indicated); all nodes have ≥ 50% support; branch length unit is substitutions per site. As expected, bacterial taxa form well-supported clades at both the phylum and family levels. Taxa are annotated for presence across the *Oncopeltus* life cycle (stage: E, egg; N, nymph; F, female; M, male; ALL: the five genera present in all life history sample types), as in Table 1. All genera are represented by a single species except the *Cyanobacteria*, where *Anthoceros formosae* is a molecular outgroup to the other two species (discussed in detail below).

To define a core microbiome profile, we considered all prokaryotic genera with >1% abundance in at least one of the four major life history sample types (untreated eggs, surface-sterilized eggs, nymphs or adults). We identified 28 distinct genera in 20 families (Fig. 5, Tables 1 and S2), of which 26 are Gram-negative, primarily represented by the *Proteobacteria* (16 genera distributed across the classes *Alpha*-, *Beta*- and *Gammaproteobacteria*) and the *Bacteroidetes* (Fig. 6). Of the two Gram-positive genera (phylum *Firmicutes*), *Enterococcus* was prevalent and abundant, while *Staphylococcus* occurred at low levels (2–3%) in only some treatments (EYW and M, Table 1). Overall, five core genera are present in all sample types: *Enterococcus, Chryseobacterium, Sphingobium, Delftia* and *Sphingobacterium* (Fig. 5a). These five accounted for one-third to fully two-thirds of the entire microbiome complement, depending on life history stage (Fig. 5b, central Venn diagram intersect). Consistent with patterns of species-level diversity (Fig. 4), egg-stage samples were comprised of up to twice as many bacterial genera as adult-stage samples, with 23 of the 28 genera present in eggs (untreated and/or surface-sterilized) compared to only 15 genera in adults (males and/or females, Fig. 5a, c).

 To better assess microbiome diversity, we phylogenetically mapped the life history stage distribution of the 28 core genera (Fig. 6). The five genera present throughout the milkweed bug life cycle are broadly distributed across the tree. The greater microbiome diversity at the egg stage chiefly arises from additional genera that are closely related to the main five. For example, all 11 genera in the clade of *Beta*- and *Gammaproteobacteria* are present at the egg stage. Except for *Delftia* being present throughout the life cycle, the other *Betaproteobacteria* – all specifically of the family *Comamonadaceae* – are restricted to embryonic samples (Fig. 6, dashed outline), and post-embryonic presence of any *Gammaproteobacteria* was patchy. Notably, no bacterial genera were uniquely present in either the surface-sterilized eggs or adult females (Fig. 5a, Table 1), so we next explored abundance dynamics of specific bacterial taxa to infer patterns of inheritance and acquisition.

### The egg surface and interior have distinct bacterial profiles

We examined egg-stage microbiome composition spatially and temporally. Greater relative abundance in untreated eggs reflects greater presence on the eggshell surface. Conversely, greater abundance in surface-sterilized samples is indicative of enrichment within the egg (yolk). We also assessed changes over time, as populations of bacterial taxa may expand or contract within the egg niche.

For both the untreated and surface-sterilized egg samples, five genera were lost and two other genera were gained in older eggs compared to young eggs (Fig. 5c, Table 1). Some of these changes likely reflect fluctuations relative to the 1% presence threshold, rather than composition turnover. However, genera with restricted presence (only in two life history stages, Table 1) may represent transient, opportunistic taxa in young egg samples (*Acidobacteria* in EY and EYW) or that proliferate on the eggshell surface (*Brevundimonas* in EO). Conversely, some genera are present in adult females and persist or thrive within eggs but are not maintained on the egg surface (*Enterobacter*, *Kosakonia* and *Klebsiella*).

**Fig. 7.**
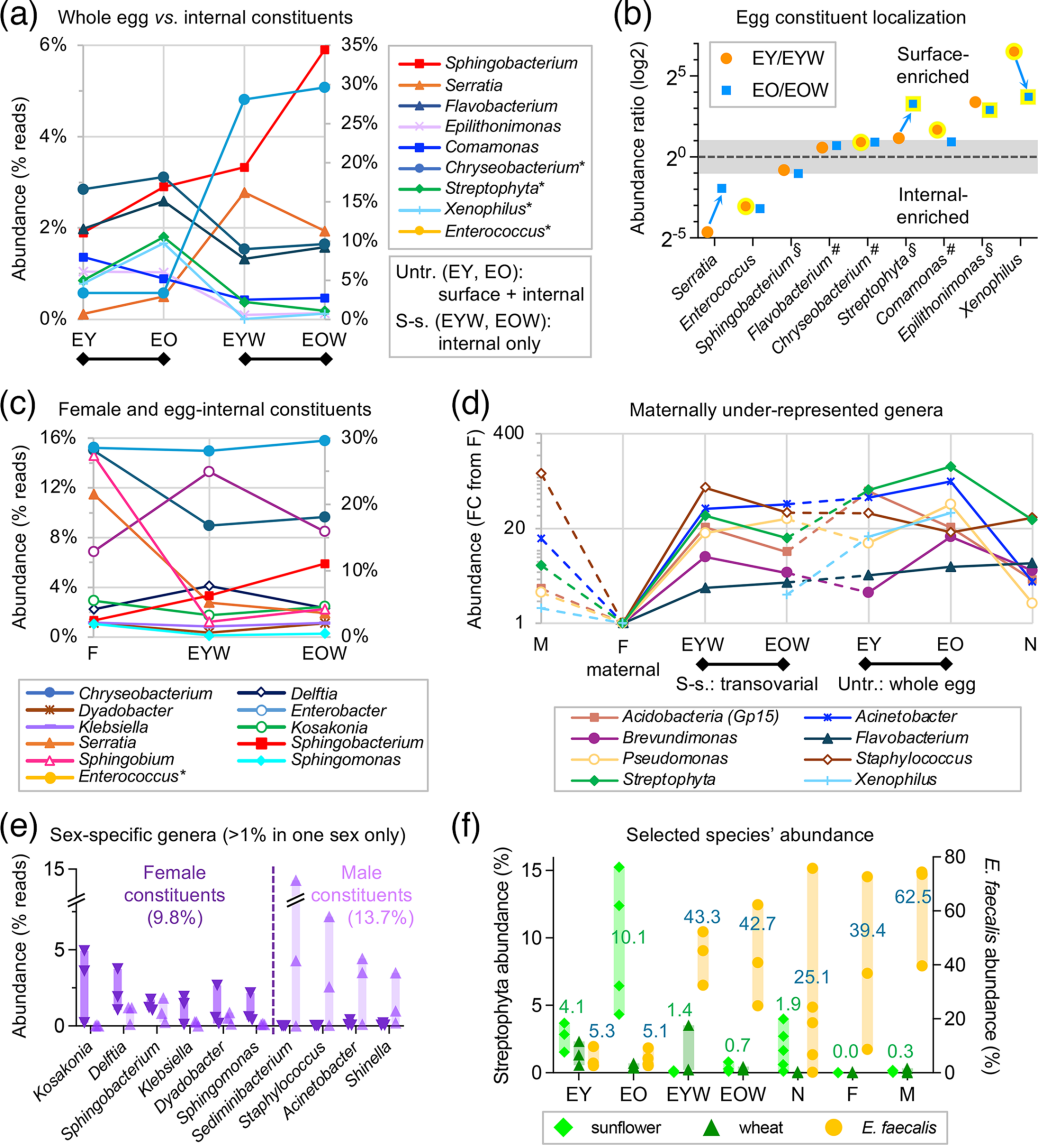
Life history stage-specific trends in bacterial abundance and transmission. Values reported per sample type are based on the mean of the biological replicates (*n*=5 for nymphs, *n*=4 for EO eggs and *n*=3 for all other samples). (a) Mean abundance for genera that differ in eggs: cool colours (green, blue and purple) indicate greater abundance in untreated eggs; warm colours (red, orange and yellow) indicate greater abundance in surface-sterilized eggs (see legend, abbreviations as in Fig. 2). Genera with high abundance are denoted by an asterisk in the legend and plotted on the right-hand y-axis. Black lines below the plot indicate temporal progression within a treatment condition. (b) Locally enriched egg constituents are depicted as stage-specific ratios of untreated to surface-sterilized samples. Large differences (>2-fold) persist even with changing relative abundance during embryogenesis (blue arrows). Yellow highlighting indicates significant differences (*p*<0.05) between untreated and surface-sterilized samples from unpaired, two-tailed comparisons; all tests were *t*-tests with Welch’s correction unless otherwise annotated: # for standard *t*-test (for EY/EYW and EO/EOW comparisons), § for non-parametric Mann-Whitney test (for EY/EYW comparisons only). (c) For the 11 genera present at >1% in adult females (F), mean relative abundance is shown in relation to transovarial transmission (EYW) and subsequent development (EOW). The genus with high abundance is denoted by an asterisk and plotted on the right-hand y-axis. (d) Eight genera are only detected at <0.5% in females yet are present in eggs (>2% in ≥1 egg samples). On the log10 scale, values <1 are not shown (for *Brevundimonas* and *Flavobacterium* in males or for *Xenophilus* in EYW and nymphs). Solid lines indicate developmental (temporal) continuity; dashed lines link samples that are not sequential. (e) Individual biological replicates (*n*=3) are plotted for the ten genera that differ in presence/absence between the sexes (adult females: dark purple, downward-pointing triangle plot points on the left; adult males: light purple, upward-pointing triangles on the right). (f) Individual biological replicates (*n*=3–5) are plotted for three selected species, as indicated in the legend. Note that species-level values differ somewhat from genus-level values (e.g., other panels of this figure, Table 1), due to differences in read classification rates (Fig. 3a). Values are mean abundance for *E. faecalis* or the sum for both sunflower and wheat as *Streptophyta* constituents.

Stably over time, we find spatial differences in enrichment: within the egg for *Serratia* and *Enterococcus* and on the egg surface for *Chryseobacterium*, *Streptophyta*, *Comamonas*, *Epilithonimonas* and *Xenophilus* (Fig. 7a, b). The latter three genera are only present at the egg stage, and the relative abundance of both *Streptophyta* and *Xenophilus* more than doubles on the egg surface during embryogenesis (Table 1). This suggests that, even in a simplified laboratory insect colony, the egg surface can foster taxa that are otherwise minimally present. On the other hand, both *Enterococcus* and *Sphingobacterium* are not only enriched within the egg (Fig. 7b) but also present throughout the milkweed bug life cycle (Fig. 5). This suggests that these taxa are transovarially transmitted as major taxa.

### The egg stage microbiome does not simply recapitulate the maternal profile

Regarding potential transovarial transmission, of the 11 genera present in females (Table 1), 10 are also detected within surface-sterilized eggs (Fig. 7c, the exception being *Sphingomonas*). However, over developmental time (across F, EYW and EOW), relative abundance is variously: stable (*Enterococcus* and *Kosakonia*), fluctuates in young eggs before returning to maternal levels in late embryogenesis (greater in EYW: *Delftia* and *Enterobacter*, lower in EYW: *Klebsiella* and *Dyadobacter*), less abundant in eggs than females (*Chryseobacterium*, *Sphingobium* and *Serratia*) or increases (*Sphingobacterium*). Thus, variable abundance dynamics argue against transmission of a specific bacterial community as a suite of correlated taxa at the maternal to embryonic life history stage transition.

Furthermore, as noted above, female bacterial taxa only represent a fraction of egg stage bacterial diversity (Figs4  5). We thus evaluated taxon-specific abundance across all life history samples for eight genera with very low female abundance (<0.5%) and >4× greater egg stage abundance (>2% in at least one egg-stage sample). Relative to adult females, all eight genera are more abundant at other life history stages (Fig. 7d), but only half of these have >1% abundance in either a nymphal or adult male sample. This suggests that some egg-stage bacterial genera are unique – neither enriched in the female reproductive tract [33,36] nor in other post-embryonic bacteria-harbouring tissues [69].

### Sex-specific differences in the adult microbiome profile

Adults have the lowest bacterial diversity of all life history stages (Fig. 4), with five shared bacterial genera accounting for the majority of the microbiome (77% in females, 70% in males, Table 1). However, fully 10–14% of the adult microbiome is sex-specific, represented by ten genera (Fig. 7e). Most are variably present, with below-threshold abundance (<1%) in at least one biological replicate. Only *Delftia* and *Sphingobacterium* were consistently present (>1%) in all female replicates – as well as in at least one male replicate, supporting their inclusion as part of the core microbiome (Fig. 5a). Despite variability between individuals, we do detect striking sex-specific differences. Compared to a scarcely detectable <0.03% in the other sex, individual males harboured up to 7% *Staphylococcus* or 14% *Sediminibacterium*, with *Kosakonia* as the major female-specific constituent at up to 5%.

### Species-level prokaryotic profiles highlight taxon proportionality and environmental components

Certain additional patterns in the *O. fasciatus* microbiome emerge in species-level analyses, with bacterial abundance differing across compared to within genera. *Chryseobacterium* is present in all life history samples (Table 1), but with high variation (range: 0.83–56.3% in post-embryonic individuals, *n*=9; see Data File S1, tab ‘Species (counts)’ for this and further details supporting this subsection). Nonetheless, species’ proportions are remarkably uniform. Consistently, the two most abundant species account for 94% of *Chryseobacterium* reads: *Chryseobacterium tructae* at 50.7±1.5% and *Chryseobacterium hominis* at 43.4±1.9% (median ±median absolute deviation, among 26 total *Chryseobacterium* species). Thus, even when a genus varies as a fraction of the total microbiome, the proportions of major species are consistent within the genus.

This pattern is corroborated by the major constituent *Enterococcus* (Figs3c and 7ac, Table 1). *Enterococcus faecalis* is the dominant species (of 13), with >97% of *Enterococcus* reads. Nonetheless, *E. faecalis* abundance is extremely variable (Fig. 7f). In individual post-embryonic insects (*n*=9), *E. faecalis* abundance ranged from an astonishing >72% of the microbiome (four individuals: one nymph, one adult female, two adult males), to <10% (two individuals), to only 0.2% in one nymph. Given our experimental design of simultaneous sampling for all individuals of a given life history stage, these are striking differences in microbiome composition at the intra-specific, within-population level.

Microbiome evaluation from a uniformly simple laboratory colony also reveals stage-specific importance of environmental components. We detect the plant chloroplast *16S rRNA* gene (phylum *Cyanobacteria*, genus *Streptophyta*, Figs3b  6). This is a known issue in microbiome profiling of phytophagous arthropods, including seed-feeding insects, and is generally regarded as contamination [70]. Yet, whole body or egg clutch material is a form of holobiont or environmental sample. In all five non-adult samples (EY, EO, EYW, EOW and N), *Streptophyta* accounted for 1–11% of genus-level reads (Table 1, Fig. 7f). This contrasts with negligible detection in any adult sample (<0.34% in five of six samples, Fig. 7f), despite uniform handling of nymphal and adult material (see the ‘Methods’ section). Moreover, *Streptophyta* is a defining constituent of untreated eggs, doubling in abundance during embryogenesis, from 5.0 to 10.5% (Fig. 7a, b, Table 1). At the species level, sunflower (*Helianthus annuus*) and wheat (*Triticum aestivum*) accounted for up to 95% of *Streptophyta* reads in all samples. Low-level detection of wheat likely does represent minor contamination from milled flour dust, from food stocks for other insect species maintained in the same laboratory rooms. In contrast, sunflower seeds are the food source for the milkweed bug colony and thus a direct environmental component. As detection of sunflower *16S* material was stage-specific but otherwise very low (see the next section), we consider this to be a specific environmental feature (see the ‘Discussion’ section).

### Representative sampling with minimal sequencing, including for environmental material

Finally, we used Sanger sequencing data in a distinct genus-level classification analysis, assessing bacterial diversity detection and clarifying environmental levels of *Streptophyta* with a wholly independent dataset and analysis method from that used in the main high-throughput dataset (see the ‘Methods’ section).

In insect samples, we detected a good balance of major, abundant taxa and rare, but genuine, constituents (Table S1). Sequencing 5–9 clones was sufficient to detect 2–6 distinct bacterial genera per sample, with detection of ≥4 genera in five of the eight samples. For example, Sanger sequencing of nymphal sample N11 recovered *Enterococcus* (66.2% in the full-scale dataset) as well as *Sphingobium* (the second-most abundant nymphal genus) and a member of the family *Enterobacteriaceae* (rare nymphal genera). Across life history samples, 94% of clones belonged to the 28 main genera (Table 1). Three clones were classified outside the main genera (<1% abundance in the main dataset), but two of these were still widely detected (*Arthrobacter* and *Acidovorax*: see Data File S1, tab ‘Genus (%, counts)’). Overall, the 50 sequenced clones identified eleven distinct bacterial genera. Thus, while small-scale sequencing clearly will not document the full scope of the microbiome, it was more effective than anticipated (cf., [33,71]) at detecting a meaningful variety of taxa.

Building on this framework, we sequenced samples taken directly from laboratory food stocks for our insect cultures, obtaining good sensitivity and accuracy. All nine clones from two biological replicates of sunflower seeds exclusively identified *H. annuus* chloroplast DNA, while all five clones from a sample of milled flour only identified chloroplasts of the grass family Poaceae (a mix of wheat and maize, Table S1). While a greater depth of sequencing of food material could potentially uncover bacterial material, our findings support the homogeneity of the laboratory environment as a controlled setting for directly assessing insect intra-individual diversity. However, natural or complex food sources of both phytophagous [59] and carnivorous [72] insects could themselves harbour diverse and variable bacterial constituents.

Notably, we did not detect any chloroplast (*Streptophyta*) material in Sanger sequencing of the insect-derived samples, which included the two biological replicates with the highest levels of *Streptophyta* in the full-scale dataset (samples EO6 and EO7, with 17.0% and 12.8% *Streptophyta*, respectively). We, therefore, conclude that stage-specific detection of sunflower material is a specific attribute of these samples, rather than the detection of general environmental material.

## Discussion

We present a nuanced account of the microbiome of the milkweed bug *O. fasciatus*, a Lygaeinae species that lacks both midgut crypts and bacteriocytes. In characterizing the core microbiome profile, we assess the implications of changing abundance between individuals and across life history stages for modes of bacterial acquisition. We also discuss filtering of very highly abundant taxa as a means of considering potentially meaningful microbial constituents per life history stage, and we present a meta-study comparison of 71 species from 5 insect orders.

### Microbiome repertoires are dynamic across the life cycle, with complex acquisition

By assessing the microbiome profile at each of the phylum, family, genus and species levels (Figs3^7^), we dissected several dynamic trends, including high bacterial diversity at the egg stage that declines post-embryonically. We also find notable differences across the life cycle for bacterial repertoires: within or external to the egg, between the sexes and for stage-specific levels of variation between individuals (Figs3b, c  7). Thus, attention to sampling and numerical handling is important for identifying patterns of bacterial abundance from noisy data, even for biological replicates derived from controlled laboratory colony environments.

Is vertical transmission predominantly transovarial, directly into the egg, or via maternal secretions onto the eggshell surface? We find conflicting evidence for the maternal bacterial repertoire. The five most abundant genera in females (each >6%) have mixed localization profiles in eggs, with three internally enriched and two externally enriched. Of the six other genera in females, four are externally enriched in young eggs – consistent with egg-smearing transmission. However, these taxa decline externally and increase internally during embryogenesis, supporting the importance of transovarial transmission. Equally, egg-stage bacterial genera exhibit conflicting patterns. Internally enriched taxa include *Enterococcus* and *Serratia*, which are characteristic of both males and females. As shared bacterial taxa in adults are few (Fig. 7e), and this is a persistent egg-stage feature, these could be key constituents. However, the absence of *Serratia* in nymphs challenges this. Aside from *Chryseobacterium* as a stable constituent across the life cycle, no other egg surface-enriched bacteria are present (>1%) in females, suggesting they are environmental constituents of the egg niche or that these taxa are vastly outcompeted within the female. Interestingly, in the pea aphid, the obligate endosymbiont *Buchnera* and the facultative endosymbiont *Serratia* (also detected in *O. fasciatus*) differ in their mode of transmission from the female to the egg [32]. Hence, the diversity of transmission routes seen across the Hemiptera [30,31] may also pertain even within a single species, belying the expectation for a global pattern.

To what extent is bacterial relative abundance important? In some cases, physiologically important bacteria are prevalent, such as *Burkholderia* symbionts in the fellow bug *Riptortus pedestris* [71]. Indeed, abundance can be a direct method of benefit to the insect host (‘colonization resistance’), such as through chemically dictating the environment (e.g. acidic or alkaline, [reviewed in 73]), or by outcompeting potentially pathogenic bacteria, as seen for *E. faecalis* in the silk moth *Bombyx mori* [reviewed in 49].

However, there need not be a correlation between abundance and biological relevance. The high levels – and large inter-individual fluctuations – that we report here in constituents like *E. faecalis* argue for targeted approaches. This could include filtering highly abundant taxa. For example, when excluding *Enterococcus* from our dataset, 75% of biological replicates (18 of 24) gained bacterial genera at >1% abundance (1–11 additional genera per sample). Bacterial diversity more than doubled for the individual nymphal and male samples that were dominated by *Enterococcus* (>56%). Across all samples, half of the gains were relative increases that reinforce the prevalence of the core 28 genera (Table 1). Among additional genera gained after filtering, all post-embryonic individuals with>56% *Enterococcus* showed an increase in the fellow Gram-positive, lactic acid bacteria *Lactobacillus* and *Vagococcus*. Gains in *Proteobacteria* included *Pseudoxanthomonas* in nymphs (closely related to the nymph-enriched core genus *Stenotrophomonas*). Intriguingly, after filtering, we also detect *Polaromonas*, another member of the egg-characteristic *Comamonadaceae* family, in both F and EYW samples, suggesting this too may be a potential transovarially transmitted constituent. Thus, filtering may reveal meaningful bacterial diversity that is otherwise concealed by the most abundant genera.

### Detection of chloroplast material: more than simple contamination

We report consistent, notable detection of chloroplast *16S* DNA from the insect’s sunflower seed food source in untreated eggs and nymphs, but not in surface-sterilized eggs or adults (Fig. 7f). Neither the spatial structure of the insect colonies (oviposition into cotton wool, remote from the food dish) nor the dormant state of the seeds (neither germinating nor dusty) accounts for specific egg-stage surface or nymphal detection. Clearly, the eukaryotic food source is not a component of the microbiome. Nonetheless, we emphasize this *16S* detection pattern as a potentially defining characteristic for how nutritional material may be sequestered (by nymphs?) or provisioned (by females at the oviposition site?) in a life history stage-specific manner (see the next section for speculation on its potential relevance).

### Comparisons with other insects’ microbiomes: shared genera and nitrogen-fixing taxa

We compared the core milkweed bug microbiome with reports of the predominant genera in other insects. Overall, *O. fasciatus* has a fairly typical insect and hemipteran microbiome profile, albeit without well-known symbionts of fellow hemipterans. Surprisingly, this is in part irrespective of feeding ecology type.

With the closely related firebug *Pyrrhocoris apterus*, 16 bacterial genera are shared (Table S2). This nuanced study on the firebug compared bacterial profiles across different geographical sites, life history stages, segments of the midgut and laboratory colony diets, as well as environmental sampling of the primary linden seed food source [59]. As in our findings in the milkweed bug, the highest levels of bacterial diversity in the firebug occurred at the egg stage, where ‘other’ genera outside of the most prevalent 15 accounted for fully a third of the microbiome profile at this stage. Furthermore, when *P. apterus* was also raised on a diet of sunflower seeds, there was a greater abundance of *Acinetobacter*, *Delftia* and *Sphingobium*, all of which are major constituents in *O. fasciatus*. In the environmental analysis of the natural linden seed food source, bacteria on the seeds included several prominent constituents in the *P. apterus* midgut. Regardless of whether this represents undigested food or part of the firebug microbiome, five of these genera are also present in the milkweed bug. In sum, a large proportion of the milkweed bug core microbiome is shared with a fellow seed-feeding bug, and a number of these bacterial taxa are closely associated with the diet.

More widely, we found microbiome commonality not only with other hemipterans but also with a carnivorous hemimetabolous insect and phytophagous holometabolous species (Table S2, 71 species in 5 insect orders [54,59, 60, 69, 73,77]). *Pseudomonas* and *Acinetobacter* occur widely across these diverse insects. While prevalent, *Enterococcus* is neither ubiquitous nor necessarily represented by *E. faecalis* (*Enterococcus mundtii* dominates in the moth *Spodoptera littoralis* [54]). Several taxa were not present in the carnivorous earwig (Dermaptera [77]) but are frequently detected across the Hemiptera and Holometabola (selected Coleoptera, Lepidoptera and Diptera), including *Serratia*, *Enterobacter*, *Klebsiella* and *Sphingomonas*. Yet, eight genera are shared between the milkweed bug and the earwig. This contrasts with limited overlap (only three genera) with either a rice-feeding leafhopper [69] or a seed-feeding stinkbug [75] as fellow phytophagous hemipterans.

Interestingly, *O. fasciatus* harbours two nitrogen-fixing, soil-associated bacteria: *Klebsiella* [78,79], widely occurring in insects, and *Rhizobium*, which may be a specific constituent of seed-feeding bugs (Tables 1 and S2 [59,75, 76]) or of milkweed plants and their natural insect herbivores [80]. In previous work on insects' capacities for nitrogen metabolism, we observed that *O. fasciatus* and other hemipterans’ genomes do not encode enzymes for arginine synthesis, and we suggested that this was obviated by consuming a nutrient-rich seed diet [11,81]. However, it is tempting to now speculate that particularly *Rhizobium* may indirectly derive nutritional benefits from association with seed-feeding insects, which could be consistent with our observations on structured detection of chloroplast material. In turn, *Rhizobium* may supply these Hemiptera with essential nitrogen metabolism components – such as for pteridine synthesis that supports *Oncopeltus*’s aposematic warning colouration [11,45] – providing nutritional symbiosis and complementation.

Finally, we considered known hemipteran nutritional endosymbionts. However, these bacterial genera were not detected or were negligible (as low as <0.008%). Specifically, we do not detect: *Burkholderia*, environmentally acquired in many stinkbugs [34,82, 83]; the kissing bug *Rhodnius prolixus*’s *Rhodococcus* [84]; or *Candidatus* endosymbionts, known in several lygaied and stinkbugs [29,39]. Unsurprisingly, we do not detect the pea aphid *Acyrthosiphon pisum*’s exclusive, obligate symbiont *Buchnera* [20]. *Wolbachia*, which provides the bed bug *Cimex lectularius* with nutritional benefits aside from its well-known, widespread influence on insect sex determination [85], is also not present in *O. fasciatus*.

In sum, this selected comparison documents 20 of the 27 milkweed bug core bacterial genera in other insects (Table S2). Five other genera were detected with low abundance in few samples. Exceptions to this are *Kosakonia*, which is prevalent across milkweed bug life history stages, and *Xenophilus*, which is notably abundant in untreated eggs (3.5–12.7% per sample) as part of the greater *Comamonadaceae* diversity revealed in our focused egg-stage microbiome sampling. Future, targeted research will help to clarify which of these bacterial taxa comprise genuine nutritional symbionts (obligate or facultative) and which are merely commensal or even pathogenic [49,73].

To explore these trends, future work should also include further rigour in experimental design, in line with increasingly prevalent standards [66,67]. As documented in the methods and supplement, we analysed DNA yields in negative controls from DNA extraction solution and library synthesis, with careful independent preparation of both technical and biological replicates, statistical assessment of potential contaminants with the *decontam* programme [68], independent sequence analysis of environmental sources from laboratory food stocks (Table S1) and appraisal of our findings compared to both the insect microbiome literature and documentation of known contaminating taxa in certain contexts (Table S2, Fig. S1 [64,65]). We also focus on robust patterns from multiple replicates and substantial abundance (e.g. Fig. 5, Table 1). Nonetheless, full-scale sequencing of the multiple negative controls and inclusion of a dilution series positive control [65,67, 68] would strengthen assessments. This would be particularly valuable regarding low biomass egg-stage microbiome diversity (Fig. 4 [59]) and considering the limits of detection for probing biologically relevant but minor constituents in post-embryonic stages (discussed above).

## Conclusions

Our controlled sampling of the microbiome from a laboratory colony of the milkweed bug documents high variability between individual insects for some bacterial taxa – a feature that has been understudied to date [discussed in 49]. We also find marked differences in the overall microbiome repertoire and diversity between life history stages, even though, as a hemimetabolous species without complete metamorphosis, *O. fasciatus* exhibits no major changes in anatomy, behaviour or ecology across the life cycle. Nonetheless, we define a robust core of 28 prokaryotic genera in *O. fasciatus*, most of which are typical for insects. As in previous work [e.g. 38, 59], direct visualization of bacteria within *O. fasciatus*, as well as tissue-specific *16S* rRNA gene profiling, will clarify where bacteria are housed and the extent to which selected taxa may be transovarially transmitted. Also of note is the prevalence of nitrogen-fixing *Rhizobium* in several Hemiptera and in *O. fasciatus* the prevalence and diversity of the *Comamonadaceae* family at the egg stage. It will be interesting to examine the extent to which these trends are borne out for *O. fasciatus* feeding on its native food source of cardenolide-containing *Asclepias* wildflower seeds in natural populations.

## Supplementary material

10.1099/mgen.0.001583 Uncited Supplementary Material 1.

10.1099/mgen.0.001583 Uncited Supplementary Material 2.
