# Supplementary material for: Intraspecific microbiome dynamics across the life cycle of the milkweed bug Oncopeltus fasciatus
Source: Microb Genom. 2026 Jan 8;12(1):001583. doi: 10.1099/mgen.0.001583 (PMC13293310; doi:10.1099/mgen.0.001583)
Supplement: Uncited Supplementary Material 2. [file mgen-12-01583-s002.pdf]

Intraspecific microbiome dynamics across the life cycle of the milkweed bug *Oncopeltus fasciatus*

Supplementary Information

In this PDF:

Figure S1. Contaminant-calling assessments.

Table S1. Sanger-sequencing results.

Table S2. Comparison of main bacterial genera across selected insect species.

Additional supplementary file:

Supplementary Data File S1. Primary data on *16S rRNA* read classification (Excel file**).**

Samples are identified by life history type (EY, EO, EYW, EOW, Ni, Np, N all, F, M: see Fig. 2) and technical replicate (K, R: see Methods), and with a unique ID number per sample.

Numerical details are provided on MiSeq and Sanger sequencing data, in 7 tabs in the file:

- Biomass and yields: per-sample tissue inputs, DNA and MiSeq library yields, and reads.
- Phylum (%, counts): Organized from left to right, the number of reads assigned to each phylum is presented for: the mean % abundance across biological replicates, the mean % abundance across technical replicates, the mean % abundance per sample, and finally the read counts per sample. The 6 most abundant phyla are highlighted in light yellow. Grey shading indicates abundance <1% (for mean % abundance, columns B:J) or 0 reads (for read counts per sample, columns CE:DZ).
- Genus (%, counts): Organized from left to right in the same manner as for the Phylum tab. The main 28 genera are highlighted in light yellow. Grey shading indicates abundance <1% (for mean % abundance, columns B:J). Red and green shading indicate <10 reads or >1000 reads, respectively (for read counts per sample, columns CE:DZ).
- Species (counts): Read counts per sample and per species are presented. Species are listed alphabetically, with highlighting for genera and species featured in the main text: *Chryseobacterium* (light yellow) and *C. tructae* and *C. hominis* (dark yellow), *Enterococcus* (light orange) and *E. faecalis* (bright orange), *Streptophyta* (light green) and *Helianthus annuus* and *Triticum aestivum* (bright green). Grey shading indicates 0 reads.
- Read depth statistics: Reports the total number of classified reads at the species level as well as the associated Shannon Species Diversity Index and total number of species identified, for all 48 samples and evaluated per technical replicate. Descriptive statistics as well as calculations and plots of correlation coefficients and t-test comparisons between technical replicates are provided. As we find no correlation between read depth and species diversity, we consider that sufficient read depth was obtained for all samples.
- Contaminant assessment: Frequency-based statistical classification of potential contaminants with the *decontam* R package (v.1.28.0).
- Sanger raw data: Identifiers and unclipped raw sequence data for all sequenced clones, supporting Table S1. Note that low quality sequences were excluded from the analysis.

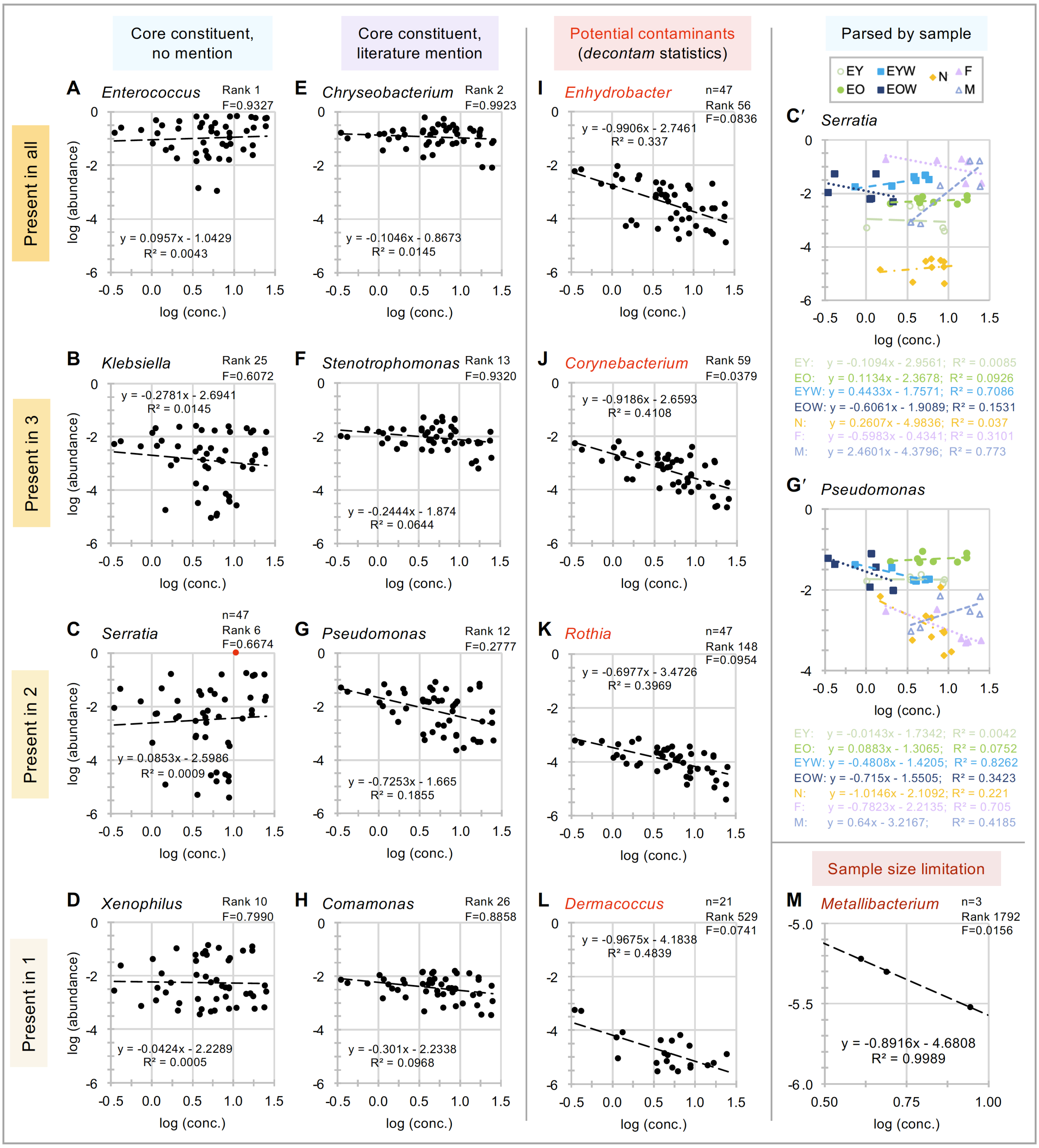

Fig. S1. Contaminant-calling assessments. Plots of the mathematical relationship underlying the *decontam* frequency-based calculation of potential contaminating bacterial genera: contaminating taxa should show an inverse (negative) relationship between library DNA concentration and taxon relative abundance, whereas non-contaminants' relative abundance should be independent from (invariant with respect to) library concentration [1]. Strong evidence of contamination is reflected in a low "F" statistic (threshold <0.1), and with a strong fit (R^2 value) of the data to a linear trendline with slope of -1 (with concentration and abundance both plotted on a log10 scale). The full statistical results are presented in Supplementary Data File S1, tabs "Biomass and yields" and "Contaminant assessment". In total, we visually inspected plots for 40 genera: all 28 core microbiome genera (Table 1), six genera flagged by the *decontam* analysis, and a further six low-abundance genera. Here, we present selected taxa to: **(A-H)** show that the core constituents of the *O. fasciatus* microbiome are statistically supported as genuine constituents, **(I-L)** confirm that the statistical threshold

is appropriate to correctly flag potential contaminant genera, including those with noisier data, **(C′-G′)** consider patterns for independent DNA extraction and PCR experiments and for biological sample types, and **(M)** depict the limitations of the frequency-based approach for small samples sizes. Numerical details in the upper right for each plot indicate the abundance rank of the specified genus (as in Supplementary Data File S1, tab "Genus (%, counts)"), the *decontam* “F” statistic, and sample size (number of individual samples in which the bacterial genus was detected: specified only if not detected in all 48 samples). Also depicted within (A-M) or below (C′-G′) each plot are the linear trendline equation and R^2 values.

(A-H) Two representative genera are shown for each of the four levels of occurrence across *O. fasciatus* life history stages (as in Table 1; rows organized by declining occurrence from “present in all” to “present in one” for the five major sample types of untreated eggs, surface-sterilized eggs, nymphs, adult males, and adult females). A number of bacterial genera have been identified as potential contaminants from various commercial DNA extraction kits [2, 3]. Although we used a different commercial kit, nonetheless our data are organized into columns for *O. fasciatus* constituents that were not identified as potential contaminants (A-D, “no mention”) and those that were named in at least one of the two studies (E-H, “literature mention”). Despite these cautionary notes from the technical literature, all 34 genera named in the main text results and discussion as *O. fasciatus* constituents strongly pass the *decontam* assessment, with “F” statistic values of >0.275. Supporting this, the plots show an absence of any strong inverse linear trend: A and C have a slight positive slope; B, D, E, F, and H all have weakly negative slopes of ≤-0.30; and all eight have an R^2 value of <0.19 (although see below for further discussion on *Pseudomonas*). From the biological literature, please also see the relative prevalence of these taxa across our meta-study of 71 insect species from 5 different orders (Table S2). Note that in one nymphal sample *Serratia* was not detected, indicated by the red plot point with abundance 0 (C), shown here for illustrative purposes but not included in the calculation of the trendline or subsequent analysis (C′); for all other taxa, samples with 0 abundance have been omitted (I, K, L, M).

(I-L) Of 2,012 total genera, only four were flagged as potential contaminants. Their plots have linear slopes of ≤-0.7 (≤-0.92 for three genera), and R^2 values of 0.34-0.48. For comparison, exemplar *decontam* contaminants have slopes of -0.9 to -1.15 and strong R^2 fit values of >0.92 ([1] and https://benjjneb.github.io/decontam/vignettes/decontam_intro.html, the supporting GitHub site, last accessed 12th October 2025). We consider the suggested default of F<0.1 to be a robust threshold for contaminant calling, as it is sensitive enough to flag *Rothia* despite a weaker inverse signal (K: negative slope of only -0.7) and to flag weakly abundant *Dermococcus* even though it is only detected in 21 of 48 samples (L). Overall, while *Enhydrobacter* and *Corynebacterium* have low but consistent detection (rank 55-60, median of 105-140 reads per sample), *Rothia* and *Dermacoccus* each have negligible abundance (rank of 148 and 529, respectively, each with a median of <30 reads per sample).

(C′-G′) The data for *Serratia* and *Pseudomonas*, core constituents present in two different life history stages, are replotted for each of the seven different life history sample types (including distinguishing young and old eggs: see legend). All sample types were prepared from independent DNA extraction experiments (same DNA kit batch), and with independent subsequent PCR amplification (independent between all sample types for the “R” technical replicate; independent between the “R” and “K” technical replicates for all sample types except EYW and EOW). For selected genera, we also inspected plots parsed by PCR amplification date and also did not find evidence of contamination (not shown).

For *Serratia*, there is no strong inverse trend that also has a strong R^2 fit for any of the samples (although EOW and F have slopes of approximately -0.6, the R^2 fit values are only

0.15 and 0.31, respectively, *cf.* I-L). Consistent with reporting in Table 1, *Serratia* shows stage-specific abundance patterns: present in adults and surface-sterilized eggs, but below-threshold (<1%) abundance in untreated eggs and essentially non-detectable levels in nymphs (<0.004% in each of the ten samples). Notably, males have medium-to-high abundance with consistent values between technical replicates but variability between individuals (between biological replicates; see Supplementary Data File S1, tab "Genus (%, counts)").

Pseudomonas was not flagged as a contaminant by *decontam*. However, as the overall plot in (G) exhibits a reasonably strong negative trend (slope of -0.73), albeit with a weak R^2 fit (0.19), we further considered this core constituent present in eggs (EY, EO, EYW, EOW). The surface-sterilized eggs do show moderate negative trends with either a strong R^2 fit (EYW) or a marked negative slope (EOW), but not both together. Also, although all EYW and EOW replicates derive from the same PCR experiment, they do not show identical concentration-abundance patterns (which would potentially reflect laboratory handling derived contamination), and abundance is comparable between technical replicates even when these differ in library concentration (see Supplementary Data File S1, tabs "Biomass and yields" and "Genus (%, counts)"). These relationships also hold for the negligibly-abundant female samples, with consistent abundance between the independently-amplified technical replicates irrespective of library concentration (two biological replicates with abundance 0.05% and one individual with abundance 0.28%). Thus, a simple linear trendline is informative with large sample sizes, but with <10 samples it becomes susceptible to individual values for either library concentration in low biomass samples (EOW) or for inter-individual variability (F samples, and see panel M). Lastly, we note that *Pseudomonas* was reported in the literature as being widespread in the microbiomes of Hemiptera and Holometabola (Table S2).

(M) In addition to the four contaminants shown in (I-L), *decontam* flagged a few further genera as potential contaminants (7-9 additional genera depending on data input normalization), but those taxa were only detected in ≤4 of the 48 samples (Supplementary Data File S1, tab "Contaminant assessment"). Exemplified by *Metallibacterium*, with only three plot points there is potential for a strong fit to a negative trendline with a slope close to -1, even though this reflects detection of only a single Illumina MiSeq read in each of 3 individual samples. The limits of low sample sizes are a known feature of *decontam* analyses ([1], and associated GitHub discussion threads), and hence we only consider as potential contaminants the four genera shown in (I-L).

Table S1. Sanger sequencing results. Taxonomic classification of *16S rRNA* amplicon data from milkweed bug life history stages and environmental samples. Sequence data and classification details are provided in Supplementary Data File S1 (tab "Sanger raw data").

Sample type and ID	# Sequences	# Genera	Genus-level integrated taxonomic classification [1], and distribution/abundance [2]
Untreated eggs (EY, EO)			
EY1	6	4	<u>Present in 3 life history samples</u>: *Acinetobacter, Klebsiella* (3)[3]<br><u>In 1</u>: *Sediminibacterium*<br><u>Below threshold</u>: *Arthrobacter* (Actinobacteria: Micrococcaceae)
EO6 [4]	9	6	<u>All</u>: *Chryseobacterium, Delftia*<br><u>In 3</u>: *Acinetobacter* (4)<br><u>In 1</u>: *Xenophilus*<br><u>Below threshold</u>: *Acidovorax*<br><u>Very rare</u>: *Kocuria* (Actinobacteria: Micrococcaceae)
EO7 [4]	5	4	<u>All</u>: *Delftia, Enterococcus*<br><u>In 3</u>: *Acinetobacter, Klebsiella* (2)
Surface-sterilized eggs (EYW, EOW)			
EYW24	6	4	<u>All</u>: *Enterococcus*<br><u>In 3</u>: *Acinetobacter*, family Enterobacteriaceae: *Klebsiella / Enterobacter* (4: likely both genera)[5]
EOW30	6	2	<u>In 3</u>: family Enterobacteriaceae: *Klebsiella / Enterobacter* (6: likely both genera)[5]
Post-embryonic samples (N, F, M)			
N11	6	3	<u>All</u>: *Enterococcus* (4), *Sphingobium*<br><u>In 3</u>: family Enterobacteriaceae: *Klebsiella / Enterobacter*
F17	6	4	<u>All</u>: *Chryseobacterium, Enterococcus* (3)<br><u>In 3</u>: family Enterobacteriaceae: *Klebsiella / Enterobacter*<br><u>In 2</u>: *Serratia*
M20	6	4	<u>All</u>: *Enterococcus* (3)<br><u>In 3</u>: *Klebsiella, Stenotrophomonas*<br><u>In 2</u>: *Serratia*
Environmental: sunflower seeds stock for milkweed bug colony [6]			
Sunflower seeds, clone 4	4	1	Chloroplast: Streptophyta / Streptophyta: *Helianthus annuus* (sunflower) (4)
Sunflower seeds, clone 5	5	1	Chloroplast: Streptophyta / Streptophyta: *Helianthus annuus* (sunflower) (5)
Environmental: husbandry flour for other insect species in the lab [6]			
Milled flour	5	All Strep-tophyta	Chloroplast: Streptophyta / Streptophyta: *Triticum aestivu*m (bread wheat) (4), Plants: grasses family Poaceae (maize: *Zea*)

Table S1 Notes:

1. "Genus-level integrated taxonomic classification" refers to consensus identification from analysis with both SILVA (SINA Aligner v1.2.12, lowest common ancestor classification method against five SILVA-hosted databases, site last accessed 28 July 2025; [4, 5]) and three GenBank databases queried by BLASTn (site last accessed 28 July 2025). Per-database classification details are provided in Supplementary Data File S1 (Excel file tab "Sanger raw data"). Sequences that could not be classified with any SILVA database were excluded as low quality.

Regarding congruence and precision, across the SILVA databases, classification was noted at the finest taxonomic resolution possible from at least one of the five databases. With BLASTn, the majority of genus-level results among the top 100 hits were considered as well as the % coverage and % nucleotide identity of the single best hit. Although BLAST results are to individual accessions and thus necessarily to the species level, we report the genus level here as being more robust [6] and consistent with our primary focus (*e.g.*, Figures 5-7

and Table 1). Where the SILVA and BLASTn outputs were congruent but SILVA only provided family-level information while the BLASTn result consistently identified the same genus among the best hits, the genus is indicated here (occurred in two cases: for EO6 Comamonadacease / *Delftia*, and for flour Poales / *Zea*).

To determine the BLASTn classification, we interrogated several GenBank databases to optimize for taxonomic resolution and accuracy. For insect-derived samples, the results shown here were robustly obtained with both the "rRNA_typestrains/ 16S_ribosomal_RNA" and "refseq_rna" databases, whereas the "nucleotide collection (nr/nt)" returned best hits to the same bacterial genus, although to a different species, which is still consistent with our genus-level reporting. For the environmental samples (laboratory food stocks for various insect colonies), results are shown from the "nucleotide collection (nr/nt)" database for the sunflower seed samples and from the "refseq_rna" database for the flour sample. For neither of the environmental samples did the "rRNA_typestrains/ 16S_ribosomal_RNA" database return a Streptophyta chloroplast hit, instead classifying the samples as belonging to the bacterial clades Cyanobacteriota or Pseudomonadota. For the sunflower samples, queries in the "refseq_rna" database consistently returned entries for the fellow asterid *Solanum verrucosum* (wild potato), but not for *Helianthus annus* itself. In contrast, for the bread wheat flour sample (see main text Methods), the "nucleotide collection (nr/nt)" database returned best hits not to *Triticum aestivum* or *Zea mays*, but rather to other – improbable – members within the same subclades of the Poales grasses family (bamboo, *Indocalamus tessellatus*, or green foxtail, *Setaria viridis*, respectively). Thus, results reported here reflect the intersection of common sense with robust BLASTn results, in light of differing sequence catalogues between the different sub-databases within the GenBank repository.

Of the 64 classified sequences, 51 sequences had the same genus-level classification with SILVA and BLASTn, 2 sequences had family-level resolution with SILVA and congruent genus-level resolution with BLASTn, 2 sequences had differing SILVA database classifications (see Note 5), and 9 sequences were classified to a different genus within the same family (see Note 5 for 8 of these). In other words, 55 sequences (86%) obtained the same classification with BLASTn and at least one SILVA database, and 8 sequences (13%) were ambiguous for one specific pairing of closely related genera that were sometimes inconsistently classified even among SILVA databases alone (see Note 5).

2. "Distribution/abundance" is indicated by the underlined text in this column, where the designations "all / in 3 / in 2 / in 1" refer to the occurrence of the identified bacterial genus across the four major milkweed bug life history sample types in Table 1. The designations "below threshold" (of 1%) and "very rare" refer to level of detection of the given genus across the entire Illumina high throughout dataset, based on the results in the Supplementary Data File S1 (tab "Genus (%, counts)").

3. Parenthetical values indicate the number of sequences, if more than one sequence was classified to the same genus.

4. Sample EO6 was analyzed in the initial insect Sanger-sequencing dataset, and EO6 and EO7 were additionally analyzed as controls for the environmental sampling analysis (see Note 6).

5. In some instances, taxonomic resolution is imprecise for distinguishing the genera *Klebsiella* and *Enterobacter*, which belong to the same family and have highly similar *16S rRNA* sequences (see also Figure 6, which uses full length sequence information and not only the V3-V4 amplicon region). In some cases, BLASTn (refseq_rna database) and the SILVA database lca_tax_rdp identified *Enterobacter* while one of the other SILVA databases

identified *Klebsiella* (one sequence each for EYW24 and EOW30). In other cases, the SILVA classification was to *Klebsiella* while the BLASTn classification was to Enterobacter (three sequences for each of EYW24 and EOW30, and one sequence for each of F17 and N11). On the other hand, across samples, eight sequences were unambiguously identified as *Klebsiella* by both SILVA and BLASTn. Based on these patterns, the comment "likely both genera" indicates that among the multiple sequences for a given sample, it is likely that some represent each of these two genera.

6. For the environmental samples, DNA was extracted and PCR performed on three biological replicates for each food source. Consistent amplicon bands were obtained with all replicates, as determined by gel electrophoresis for these samples in parallel with new PCR amplification of DNA template for insect samples (EO6: new PCR of the original DNA used in Illumina sequencing, EO7: an additional, Sanger-only biological replicate) as positive controls. From these, Sanger sequencing was then performed for two biological replicates of sunflower seed material and for a single sample of milled flour.

Table S2. Comparison of bacterial genera shared between *O. fasciatus* and selected insect species. Bacterial profiles are based on the major bacterial genera reported in main text display elements of the cited studies, unless otherwise noted [7-15]. Streptophyta (chloroplast) material is not considered here.

				Insect order, feeding ecology type:	Hemimetabolous (Dermaptera), carnivorous	Hemiptera, seed-feeding	Hemiptera, seed-feeding	Hemiptera, rice plant feeding	Hemiptera, meta study (aquatic, phytophagous, carnivorous)	Holometabolous (Coleoptera), phytophagous	Holometabolous (Lepidoptera), phytophagous	Holometabolous (Lepidoptera), meta study	Holometabolous (Diptera), phytophagous	Summary of bacterial genus presence (9 cited studies: 71 species in 5 insect orders, in addition to *O. fasciatus*)		
Count	*O. fasciatus* life cycle distribution[1]	Bacterial family in *O. fasciatus*	Bacterial genus in *O. fasciatus*	Study details:	Maritime earwig, *Anisolabis maritima* (Greer 2020). 30 genera, multiple stages.[3]	Red firebug, *Pyrrhocoris apterus* (Sudakaran 2012). 15-25 genera, multiple factors.[4]	Stinkbug, *Pachygrontha antennata* (Kang 2019). 27 genera.	Leafhopper, *Recilia dorsalis* (Huang 2023). 10 genera, specific tissues.[5]	Meta study, 30 hemipteran species (Li 2022). 30 genera.	Coffee berry borer, *Hypothenemus hampei* (Mejía-Alvarado 2021). 35 genera.	Cotton leafworm, *Spodoptera littoralis* (Chen 2016). 13-16 genera, multiple stages.[8]	Meta study, 30 lepidopteran species (Paniagua Voirol 2018). 26 genera.	Fruit fly family Tephritidae, 5 species (Augustinos, 2019). 13 genera.	Presence in selected studies	Presence in selected hemipteran studies	Taxonomic distribution summary
1	Present in 2	Pseudomonadaceae	Pseudomonas			Pseudomonas (gut region, site)	Pseudomonas	Pseudomonas	Pseudomonas	Pseudomonas	Pseudomonas (E, A)	Pseudomonas	Pseudomonas	8	4	all Hemiptera and Holometabola
2	Present in 3[2]	Moraxellaceae	Acinetobacter		Acinetobacter	Acinetobacter (life cycle, site, diet-SFS)			Acinetobacter	Acinetobacter	Acinetobacter (E, A)	Acinetobacter	Acinetobacter	7	2	widespread in insects
3	Present in all	Enterococcaceae	Enterococcus		Enterococcus	Enterococcus (life cycle, site, diet)			Enterococcus	Enterococcus	Enterococcus (E, L, A)	Enterococcus		6	2	widespread in insects
4	Present in 2	Yersiniaceae	Serratia			Serratia (diet)			Serratia	Serratia	Serratia (A)	Serratia	Serratia	6	2	some Hemiptera, all Holometabola
5	Present in 3[2]	Enterobacteriaceae	Enterobacter			Enterobacter (life cycle, site)		Enterobacter	Enterobacteriaceae[6]			Enterobacter	Enterobacter	5	3	some Hemiptera and Holometabola
6	Present in 3[2]	Enterobacteriaceae	Klebsiella			Klebsiella (life cycle, site)				Klebsiella	Klebsiella (E, A)	Klebsiella	Klebsiella	5	1	some Hemiptera, all Holometabola
7	Present in 2	Sphingomonas	Sphingomonas			Sphingomonas (gut region, site, diet)	Sphingomonas		Sphingomonas		Sphingomonas (L)	Sphingomonas		5	3	some Hemiptera and Holometabola
8	Present in 3[2]	Lysobacteraceae	Stenotrophomonas		Stenotrophomonas	Stenotrophomonas		Stenotrophomonas		Stenotrophomonas		Stenotrophomonas		5	2	widespread but patchy in insects
9	Present in 3[2]	Flavobacteriaceae	Flavobacterium		Flavobacterium	Flavobacterium (gut region)					Flavobacterium (E, L)	Flavobacterium		4	1	widespread but patchy in insects
10	Present in all	Weeksellaceae	Chryseobacterium		Chryseobacterium	Chryseobacterium (diet)						Chryseobacterium		3	1	widespread but patchy in insects
11	Present in 1	Comamonadaceae	Comamonas		Comamonas						Comamonas (E)	Comamonas		3	0	patchy
12	Present in 2	Rhizobiaceae	Rhizobium			Rhizobium (gut region, diet)	Rhizobium		Rhizobiaceae[6]					3	3	Hemiptera only
13	Present in all	Sphingobacteriaceae	Sphingobacterium		Sphingobacterium					Sphingobacterium	Sphingobacterium (E)			3	0	patchy
14	Present in 2	Staphylococcaceae	Staphylococcus			Staphylococcus (diet)			Staphylococcus[7]		Staphylococcus (A)	Staphylococcus		3	1	patchy
15	Present in 2	Caulobacteraceae	Brevundimonas			Brevundimonas (gut region)						Brevundimonas		2	1	limited
16	Present in all	Comamonadaceae	Delftia			Delftia (diet-SFS)					Delftia (E)			2	1	limited
17	Present in 3[2]	Spirosomataceae	Dyadobacter		Dyadobacter									1	0	limited
18	Present in 1	Rhizobiaceae	Shinella			Shinella (diet)								1	1	limited
19	Present in all	Sphingomonadaceae	Sphingobium			Sphingobium (site, diet-SFS)								1	1	limited
20	Present in 1	Chitinophagaceae	Sediminibacterium									Sediminibacterium[9]		1	0	limited
21	Present in 2	Acidobacteriaceae	Acidobacteria											0	0	not reported / not detected
22	Present in 1	Weeksellaceae	Epilithonimonas											0	0	
23	Present in 3[2]	Enterobacteriaceae	Kosakonia											0	0	
24	Present in 1	Sphingobacteriaceae	Nubsella											0	0	
25	Present in 1	Bacteriovoracaceae	Peredibacter											0	0	
26	Present in 1	Comamonadaceae	Pseudorhodoferax											0	0	
27	Present in 1	Comamonadaceae	Xenophilus											0	0	
				# shared genera with *O. fasciatus*:	8	16	3	3	8	7	11	14	5			

Table S2 Notes:

1. See main text Table 1 for numerical details supporting bacterial distribution across the *O. fasciatus* life cycle (note that *Streptophyta* chloroplast material is excluded here). Bacterial taxa are colored by presence/distribution for the five ubiquitous genera in *O. fasciatus* (orange) or for limited presence in either the milkweed bug or the other species from the cited literature (light red). Bacterial taxa are ordered by descending prevalence across this comparison (see the final three columns of the table). Shared bacterial genera are named in the corresponding table cell; blank cells indicate the given bacterial genus was not found in the given study.

2. "Present in 3" comprises both untreated and surface-sterilized eggs as well as one post-embryonic stage.

3. *Anisolabis maritima*: 30 total bacterial, based on analysis of multiple maternal and embryonic samples.

4. *Pyrrhocoris apterus*: based on 15 bacterial genera in midgut regions M1/2/3/4; 15 genera across the life cycle (eggs, each of five nymphal instars, adult males, adult females); 25 bacterial genera from 5 geographical sites; 22 genera from 3 diets (including sunflower seeds, SFS). Where the sunflower seed diet increased the abundance of a given bacterial genus, this is indicated in the table. Among the bacterial genera shared with *O. fasciatus* are three detected on naturally occurring linden seeds and in the firebug midgut (Brevundimonas, Rhizobium, Pseudomonas) as well as two specifically on linden seeds (Stenotrophomonas and Sphingomonas).

5. *Recillia dorsalis*: sampling from targeted postembryonic tissues: midgut, salivary gland, Malpighian tubules, gonads.

6. Classification to the family level for this taxon.

7. Reported in this meta study specifically for the blood-feeding assassin bug *Sycanus croceovittatus*. Other bacterial taxa listed here were reported more widely across species.

8. *Spodoptera littoralis*: 13 bacterial genera in eggs (E), 13 genera in larvae (L), 16 genera in adults (A).

9. Lepidopteran meta study of 30 species: Bacterial genus *Sediminibacterium* was reported in the supplement for a single species (the eastern spruce budworm, *Choristoneura fumiferana*); all other genera listed in this column were present in ≥3 different lepidopteran species.

Supplementary References:

1. Davis NM, Proctor DM, Holmes SP, Relman DA, Callahan BJ: Simple statistical identification and removal of contaminant sequences in marker-gene and metagenomics data. *Microbiome* 2018, 6:226.
2. Glassing A, Dowd SE, Galandiuk S, Davis B, Chiodini RJ: Inherent bacterial DNA contamination of extraction and sequencing reagents may affect interpretation of microbiota in low bacterial biomass samples. *Gut Pathog* 2016, 8:24.
3. Salter SJ, Cox MJ, Turek EM, Calus ST, Cookson WO, Moffatt MF, Turner P, Parkhill J, Loman NJ, Walker AW: Reagent and laboratory contamination can critically impact sequence-based microbiome analyses. *BMC Biol* 2014, 12:87.
4. Quast C, Pruesse E, Yilmaz P, Gerken J, Schweer T, Yarza P, Peplies J, Glöckner FO: The SILVA ribosomal RNA gene database project: improved data processing and web-based tools. *Nucleic Acids Res* 2013, 41:D590-596.

5. Pruesse E, Peplies J, Glöckner FO: SINA: Accurate high-throughput multiple sequence alignment of ribosomal RNA genes. *Bioinformatics* 2012, 28:1823-1829.
6. Hackmann TJ: Setting new boundaries of 16S rRNA gene identity for prokaryotic taxonomy. *Int J Syst Evol Microbiol* 2025, 75:006747.
7. Greer JA, Swei A, Vredenburg VT, Zink AG: Parental care alters the egg microbiome of maritime earwigs. *Microb Ecol* 2020, 80:920-934.
8. Sudakaran S, Salem H, Kost C, Kaltenpoth M: Geographical and ecological stability of the symbiotic mid-gut microbiota in European firebugs, Pyrrhocoris apterus (Hemiptera, Pyrrhocoridae). *Mol Ecol* 2012, 21:6134-6151.
9. Kang JY, Kwon YS, Jeong G, An I, Park S: Characteristics of Microbial Communities of *Pachygrontha antennata* (Hemiptera: Pachygronthidae) in Relation to Habitat Variables. *Int J Environ Res Public Health* 2019, 16:4668.
10. Huang Q, Shan HW, Chen JP, Wu W: Diversity and dynamics of bacterial communities in the digestive and excretory systems across the life cycle of leafhopper, *Recilia dorsalis*. *Insects* 2023, 14:545.
11. Li G, Sun J, Meng Y, Yang C, Chen Z, Wu Y, Tian L, Song F, Cai W, Zhang X, Li H: The impact of environmental habitats and diets on the gut microbiota diversity of true bugs (Hemiptera: Heteroptera). *Biology (Basel)* 2022, 11:1039.
12. Mejía-Alvarado FS, Ghneim-Herrera T, Góngora CE, Benavides P, Navarro-Escalante L: Structure and dynamics of the gut bacterial community across the developmental stages of the coffee berry borer, *Hypothenemus hampei*. *Front Microbiol* 2021, 12:639868.
13. Chen B, Teh BS, Sun C, Hu S, Lu X, Boland W, Shao Y: Biodiversity and activity of the gut microbiota across the life history of the insect herbivore *Spodoptera littoralis*. *Sci Rep* 2016, 6:29505.
14. Paniagua Voirol LR, Frago E, Kaltenpoth M, Hilker M, Fatouros NE: Bacterial symbionts in Lepidoptera: their diversity, transmission, and impact on the host. *Front Microbiol* 2018, 9:556.
15. Augustinos AA, Tsiamis G, Cáceres C, Abd-Alla AMM, Bourtzis K: Taxonomy, diet, and developmental stage contribute to the structuring of gut-associated bacterial communities in tephritid pest species. *Front Microbiol* 2019, 10:2004.
